# Soluble Non-Starch Polysaccharides From Plantain (*Musa x paradisiaca* L.) Diminish Epithelial Impact of *Clostridioides difficile*

**DOI:** 10.3389/fphar.2021.766293

**Published:** 2021-12-10

**Authors:** Hannah L. Simpson, Carol L. Roberts, Louise M. Thompson, Cameron R. Leiper, Nehana Gittens, Ellie Trotter, Carrie A. Duckworth, Stamatia Papoutsopoulou, Fabio Miyajima, Paul Roberts, Niamh O’Kennedy, Jonathan M. Rhodes, Barry J. Campbell

**Affiliations:** ^1^ The Henry Wellcome Laboratories of Molecular & Cellular Gastroenterology, Faculty of Health & Life Sciences, University of Liverpool, Liverpool, United Kingdom; ^2^ Department of Clinical Infection, Microbiology and Immunology, Institute of Infection Veterinary and Ecological Sciences, Faculty of Health & Life Sciences, University of Liverpool, Liverpool, United Kingdom; ^3^ Department of Biochemistry and Biotechnology, School of Health Sciences, University of Thessaly, Larissa, Greece; ^4^ Wolfson Centre for Personalised Medicine, Department of Molecular & Clinical Pharmacology, Institute of Systems, Molecular & Integrative Biology, University of Liverpool, Liverpool, United Kingdom; ^5^ Oswaldo Cruz Foundation (Fiocruz), Eusébio, Brazil; ^6^ Department of Microbiology, Liverpool Clinical Laboratories, Royal Liverpool and Broadgreen University Hospitals NHS Trust, Liverpool, United Kingdom; ^7^ School for Medicine and Clinical Practice, University of Wolverhampton, Wolverhampton, United Kingdom; ^8^ Provexis PLC, c/o The University of Aberdeen, Aberdeen, United Kingdom

**Keywords:** *Clostridioides difficile*, spores, toxin, intestine, epithelium, soluble dietary fibre

## Abstract

*Clostridioides difficile* infection (CDI) is a leading cause of antibiotic-associated diarrhoea. Adhesion of this Gram-positive pathogen to the intestinal epithelium is a crucial step in CDI, with recurrence and relapse of disease dependent on epithelial interaction of its endospores. Close proximity, or adhesion of, hypervirulent strains to the intestinal mucosa are also likely to be necessary for the release of *C. difficile* toxins, which when internalized, result in intestinal epithelial cell rounding, damage, inflammation, loss of barrier function and diarrhoea. Interrupting these *C. difficile*-epithelium interactions could therefore represent a promising therapeutic strategy to prevent and treat CDI. Intake of dietary fibre is widely recognised as being beneficial for intestinal health, and we have previously shown that soluble non-starch polysaccharides (NSP) from plantain banana (*Musa* spp.), can block epithelial adhesion and invasion of a number of gut pathogens, such as *E. coli* and Salmonellae. Here, we assessed the action of plantain NSP, and a range of alternative soluble plant fibres, for inhibitory action on epithelial interactions of *C. difficile* clinical isolates, purified endospore preparations and toxins. We found that plantain NSP possessed ability to disrupt epithelial adhesion of *C. difficile* vegetative cells and spores, with inhibitory activity against *C. difficile* found within the acidic (pectin-rich) polysaccharide component, through interaction with the intestinal epithelium. Similar activity was found with NSP purified from broccoli and leek, although seen to be less potent than NSP from plantain. Whilst plantain NSP could not block the interaction and intracellular action of purified *C. difficile* toxins, it significantly diminished the epithelial impact of *C. difficile*, reducing both bacteria and toxin induced inflammation, activation of caspase 3/7 and cytotoxicity in human intestinal cell-line and murine intestinal organoid cultures. Dietary supplementation with soluble NSP from plantain may therefore confer a protective effect in CDI patients by preventing adhesion of *C. difficile* to the mucosa, i.e. a “contrabiotic” effect, and diminishing its epithelial impact. This suggests that plantain soluble dietary fibre may be a therapeutically effective nutritional product for use in the prevention or treatment of CDI and antibiotic-associated diarrhoea.

## 1 Introduction

*Clostridioides difficile* is a highly infectious, Gram-positive, spore-forming anaerobe (Bartlett, 2010) that is recognised as the leading cause of antibiotic-associated diarrhoea, attributing 15–25% of all cases (Ananthakrishnan, 2011). *C. difficile* infection (CDI) causes a spectrum of intestinal disease, with symptoms ranging from mild diarrhoea to severe pseudomembranous colitis (PMC), bowel perforation and toxic megacolon, which can ultimately be fatal (Rupnik et al., 2009).

Adhesion of *C. difficile* to the intestinal epithelium represents an initial and crucial step in the pathogenesis of CDI (Janoir, 2016). To mediate this process, *C. difficile* possesses adhesins which exhibit carbohydrate-binding (lectin) activity. These surface adhesins recognise and bind to complementary carbohydrates on the surface of host cells, promoting bacterial-epithelial adherence (Sharon, 2006). Indeed, close proximity or adhesion of *C. difficile* to the intestinal mucosa is also likely to be necessary for the release of *C. difficile* toxins; single-chain A and B toxins (TcdA and TcdB) and the binary toxin (*C. difficile* transferase; CDT). It is well established that following their internalisation into cells, *via* clathrin-mediated endocytosis (Papatheodorou et al., 2010), *C. difficile* TcdA and TcdB are involved in mono-glucosylation of small Rho family GTPases (Rho, Rac1 and Cdc42), preventing their interaction with downstream regulators (Just et al., 1995a; Just et al., 1995b; Sehr et al., 1998). This results in their subsequent inactivation, leading to increased cell rounding, loss of actin stress fibres and disruption of intercellular tight junctions (Voth and Ballard, 2005). Other cellular effects of both TcdA and TcdB, include the induction of an inflammatory response, mediated *via* activation of nuclear factor kappa B (NF-κB) and subsequent release of the key pro-inflammatory cytokines, such as interleukin 8 (IL-8) and interleukin 1 beta (IL-1β) (Kim et al., 2006; Cowardin et al., 2016). TcdA and TcdB are also known to mediate programmed cell death through the activation of both intrinsic and extrinsic apoptotic pathways, and subsequent activation of executioner caspase-3 (Gerhard et al., 2008). *C. difficile* CDT is associated with hypervirulent strains, that may increase CDI severity and are linked to higher rates of fatal illness (Bacci et al., 2011; Cowardin et al., 2016; Berry et al., 2017; López-Cárdenas et al., 2021).

CDT is part of the family of ADP-ribosylating toxins that when cleaved binds to a lipolysis stimulated lipoprotein receptor (LSR) on the apical surface of intestinal epithelial cells (Gerding et al., 2014). The binary toxin itself consists of two unlinked polypeptides, enzymatic component CDTa and transporter component CDTb. CDTa is a mono ADP-ribosyl transferase which becomes internalised into the host, most likely *via* a CDTb heptamer on the cell surface. The internalised complex then translocates *via* receptor-mediated endocytosis to acidic endosomes where CDTb, following a decrease of the endosomal pH, creates a pore in the membrane allowing translocation of CDTa into the cytosol. There CDTa acts to irreversibly ADP-ribosylate monomeric G-actin which leads to inhibition of polymerisation to F-actin, thus disrupting the actin cytoskeleton resulting in cell rounding and cell death (Carman et al., 2011; Davies et al., 2011; Beer et al., 2018). Studies have also suggested that CDT may increase bacterial adherence to target cells through the formation of microtubule protrusions (Schwan et al., 2009; Abt et al., 2016). *C. difficile* toxins also display lectin activity, binding to specific carbohydrate moieties on the epithelial cell surface to facilitate their uptake *via* receptor-mediated endocytosis. Studies have demonstrated the direct binding of *C. difficile* TcdA to glycans expressing the disaccharide Galβ1-4GlcNAc (Tucker and Wilkins, 1991; Greco et al., 2006).

In recent years, the incidence of CDI has rapidly increased, and the disease is becoming associated with higher rates of morbidity and mortality (Rupnik et al., 2009; Freeman et al., 2010). A number of hypervirulent *C. difficile* variants have also emerged, of most note, the BI/NAP1/027 hypervirulent strain (Rupnik et al., 2009; O'Connor et al., 2009), shown to secrete elevated levels of both TcdA and TcdB (Loo et al., 2005; McDonald et al., 2005). In addition, there has been considerable increase in recurrence and relapse rates associated with CDI (Kelly, 2012), due to the ability to produce endospores (Burns et al., 2011). This robust, dormant form of *C. difficile* can survive almost indefinitely outside the host, and persists on clinical surfaces for long periods of time (Burns et al., 2011). Once ingested by a susceptible individual, spores return to vegetative growth through germination, resulting in colonisation, toxin release and subsequent active disease (Burns et al., 2011). It has also been demonstrated that *C. difficile* spores are a key vehicle of transmission in CDI, interacting with the intestinal epithelium (Paredes-Sabja and Sarker, 2012) and mucosal macrophages (Paredes-Sabja et al., 2012), leading to persistence within the intestinal tract.

It is clear that *C. difficile* bacterium, toxin and spore interaction with the intestinal epithelium all play a key role in the pathogenesis of CDI. Interrupting these bacteria-epithelial interactions could therefore represent a promising therapeutic strategy to prevent and treat CDI. Our own recent studies have demonstrated that soluble dietary plant non-starch polysaccharides (NSP), particularly their acidic (pectin-rich) components, from plantain bananas (*Musa* spp.), can block epithelial adhesion of a number of other gut pathogens (including *Salmonella enterica* serovar Typhimurium, *Shigella sonnei* and various *Escherichia coli* pathovars) to human intestinal epithelial cell-lines *in vitro* (Martin et al., 2004; Roberts et al., 2010; Roberts et al., 2013), and also block bacterial translocation across *ex vivo* human ileal explants of villous or follicle-associated epithelium (FAE) (Roberts et al., 2010; Roberts et al., 2013). The effect is mediated by interaction between the plantain pectin-rich NSP and the gut epithelial cells. Efficacy was confirmed *in vivo* in chickens, where supplementation of a commercial diet with plantain NSP was shown to block *Salmonella* colonisation (Parsons et al., 2014; Parsons et al., 2015). The purpose of the present study was to assess the action of plantain NSP on a range of *C. difficile* clinical isolates of differing PCR ribotype and toxin status, as well as testing a range of alternative soluble plant fibres for their inhibitory action on epithelial interactions of *C. difficile*. Plantain NSP was also tested for its ability to inhibit epithelial adhesion of *C. difficile* endospores, and on the cellular interactions of purified *C. difficile* toxins.

## 2 Materials and Methods

### 2.1 Human Intestinal Epithelial Cell Cultures

The human colon adenocarcinoma cell-lines Caco-2 (ECACC #86010202), SW620 (ECACC #87051203) and SW480 (ECACC #87092801) were obtained from the European Collection of Authenticated Cell Cultures (Public Health Laboratory Service; Wiltshire, United Kingdom). Intestine-407 cells (ATCC #CCL-6) were obtained from the American Type Culture Collection (LGC Standards: Teddington, United Kingdom). All cell-lines were initially grown in T-75 tissue culture flasks until 80% confluent. Cells were trypsinized, and re-seeded at 1 × 10^5^ cells/well to 24-well tissue culture plates (Corning Costar, High Wycombe, United Kingdom) for infection assays. For luminescence assays, cells were seeded at 9 × 10^4^ per well in 96-well white bottom culture plates (Costar). Parallel cultures in 96-well clear bottom plates were used to assess confluency by light microscopy. All cell-lines were cultured in complete Dulbecco’s Modified Eagle’s Medium (DMEM) supplemented with 10% v/v foetal bovine serum (Invitrogen; Paisley, United Kingdom), 100 U/ml penicillin, 100 μg/ml streptomycin and 8 mM glutamine (Sigma Aldrich; Poole, United Kingdom), and maintained at 37°C in a humidified atmosphere of 5% CO_2_/95% air for 24–48 h.

### 2.2 Murine Intestinal Organoid Cultures

Intestinal organoids were utilised to assess epithelial cytotoxicity in response to *C. difficile* purified toxins, as per (Jones et al., 2019). Mice (C57BL/6J strain, at 10-weeks old) purchased from Charles River Ltd. (Margate, United Kingdom) and were maintained for 2 weeks prior to tissue harvesting on a standard CRM pelleted rodent chow diet (Special Diet Services; Witham, United Kingdom) with water available *ad libitum*, in a specific-pathogen-free environment at the Biomedical Services Unit (University of Liverpool). Mice were sacrificed, under Schedule 1 of the Animals (Scientific Procedures) Act 1986, by rising CO_2_ levels, followed with cervical dislocation. Small intestine was dissected and transported on ice in sterile phosphate-buffered saline (PBS) pH 7.3, for use in establishing organoid cultures over 6 days, as previously described (Jones et al., 2019). On day 7, cultures were passaged by mechanically disruption through a 27G needle, resuspended in fresh Matrigel and plated out again to 24-well plates. Experimental treatment of organoids was only performed following a minimum of 1 passage.

### 2.3 Plant Soluble Non-Starch Polysaccharides

Plantain (*Musa x paradisiaca* L.) non-starch polysaccharide (NSP) preparations from excipient-free plantain flour (Trobana Green Plantain flour AAB group (Horn)); Confoco International Ltd.; Ripley, United Kingdom) were prepared by Provexis plc (Reading, United Kingdom) at the Teagasc Food Research Centre (Moorepark, Ireland) as previously described (Roberts et al., 2010; Roberts et al., 2013). A range of other NSP were also prepared, selected to include representatives of dicots, commelinoid monocots and non-commelinoid monocots. This selection ensured a wide variety of NSP composition. The plants selected included those from the families Rosaceae—apple, pear and strawberry (dicots); Ericaceae—blueberry (dicot); Musaceae—plantain (green) (commelinoid monocot); Solanaceae—tomato and bell pepper (dicots); Poaceae—oat (commelinoid monocot); Fabaceae—runner bean (dicot); Brassicaceae—broccoli (dicot); Apiaceae—celery (dicot); and Amaryllidaceae—leek (non-commelinoid monocot). All were prepared to the same specification (Provexis plc); see Supplementary Table S1. Soluble NSP concentrations tested were within the range of effective luminal concentrations in the human distal colon that would be readily achievable with dietary supplementation (Roberts et al., 2010). Neutral and acidic polysaccharide fractions of soluble NSP from plantain were prepared by Q-Sepharose anion-exchange chromatography, as per (Parsons et al., 2014).

### 2.4 *Clostridioides difficile* Strains and Growth Conditions

The laboratory *C. difficile* reference strain 1470 (ATCC #43598) was obtained from the American Tissue Type Collection (LGC Standard; Teddington, United Kingdom). *C. difficile* clinical laboratory isolate 080042 (PCR ribotype 027) used in initial intestinal epithelial cell interaction assays was obtained from Dr Godfrey Smith (Department of Medical Microbiology, Royal Liverpool and Broadgreen University Hospitals NHS Trust, United Kingdom). *C. difficile* 1342, a pathogenicity locus (PaLoc) negative strain, was provided by Drs Gillian Douce and Anthony Buckley (Institute of Infection, Immunity and Inflammation, University of Glasgow, United Kingdom), a strain having been originally obtained from Dr Kate Dingle (Oxford University, Oxford, United Kingdom) (Stoesser et al., 2011). Eleven additional *C. difficile* clinical isolates of differing toxin status and PCR ribotype, and from various geographical locations, were also obtained from the Royal Liverpool and Broadgreen University Hospitals NHS Trust; see Table 1. All isolates were initially grown in the laboratory under anaerobic conditions, at 37°C for 48 h, on Brain Heart Infusion (BHIS) agar supplemented with 0.1% w/v L-cysteine and 5 mg/ml yeast extract. Prior to infection of cultured intestinal epithelial cells, *C. difficile* strains were grown on Fastidious anaerobic agar (Oxoid), washed three times in sterile PBS pH 7.3, and resuspended to an OD_550nm_, equivalent to 1 × 10^9^ CFU/ml.

**TABLE 1:** *C. difficile* strains used.

*C. difficile* strain designation	Toxin A	Toxin B	Binary toxin	Ribotype	Geographic isolation
80042	+	+	+	027	Liverpool
98011	+	+	+	027	Liverpool
108536	+	+	+	027	Japan
98078	+	+	+	078	Liverpool
98231	+	+	+	078	Liverpool
108526	+	+	−	018	Japan
108906	+	+	−	012	Malawi
98359	+	+	−	106	Liverpool
1470	−	+	−	017	Belgium
108963	−	+	−	017	Liverpool
108519	−	+	−	017	Japan
108520	−	+	−	368	Japan
98220	−	−	−	010	Liverpool
1342	−	−	−	005	Oxford

### 2.5 Spore Isolation and Purification

Spores from five selected *C. difficile* clinical isolates (98011, 108536, 108906, 98220, and 1342) were prepared according to methods previously described (Permpoonpattana et al., 2011), with some modifications. Briefly, for each isolate, a single bacterial colony was selected from BHIS agar anaerobic culture (at 37°C for 48 h) and used to inoculate 10 ml TGY broth (3% w/v tryptic soy broth, 2% w/v glucose, 1% w/v yeast extract, 0.1% w/v L-cysteine) After overnight incubation in static conditions, TGY culture medium was sub-cultured (1:100) into Sorbitol MacConkey (SMC) broth (9% w/v/peptone, 0.05% w/v proteose-peptone, 0.1% w/v (NH_4_)_2_SO_4_, 0.15% w/v/Tris, 0.1% w/v L-cysteine) and incubated at 37°C to an OD_600nm_ = 0.5. Aliquots of culture broth were used to inoculate a dozen SMC agar plates per strain, which were incubated under anaerobic conditions, at 37°C for 10 days. After day 10, spores were harvested and washed twice with sterile water, then suspended in 2 ml sterile PBS pH 7.3 containing 125 mM Tris, 200 mM EDTA, 0.3 mg/ml proteinase K and 1% v/v sarcosyl, and incubated at 37°C for 2 h with gentle shaking. Spores were then centrifuged (4,500 x *g* for 20 min) and the pellet resuspended in cold sterile distilled water. This was repeated a further 10 times. After the final wash, spores were heat-treated (60°C, for 20 min) to kill any residual vegetative cells, and then frozen at −20°C until use. To calculate spore numbers, aliquots were serially diluted in sterile PBS and plated onto BHIS agar supplemented with 0.1% w/v sodium taurocholate for spore germination (Sorg and Sonenshein, 2008; Burns and Minton, 2011). After 48 h, spore titres were enumerated by enumerating vegetative bacteria colony forming units (CFU). To confirm purity of each spore preparation, a Schaeffer and Fulton endospore stain was routinely performed (Schaeffer and Fulton, 1933). Results indicated that the purification process effectively and reproducibly yielded spore titres of a high purity, effectively free of vegetative cells (see Supplementary Figure S1; Supplementary Table S2).

### 2.6 Soluble Fibre Blockade of *Clostridioides difficile*-Intestinal Epithelial Cell Adhesion

Confluent Caco-2 cell monolayers in 24-well plates were pre-treated for 30 min with or without soluble NSP from plantain (0–20 mg/ml in antibiotic-free DMEM). Cell monolayers were then infected with vegetative *C. difficile* clinical isolates or purified *C. difficile* spore preparations (1 × 10^7^ CFU/ml), at a multiplicity of infection (MOI) of 100, for 2 h. Each monolayer was then washed three times with sterile PBS to remove non-adherent bacteria or spores, and monolayers were lysed with sterile 1% v/v Triton X-100. Epithelial adherent vegetative *C. difficile* and *C. difficile* spores were both enumerated by performing serial dilutions of cell lysates, plating of 20 µl aliquots of lysate to BHIS agar (inclusive of 0.1% w/v sodium taurocholate for spores) in triplicate. Plates were then incubated for 48 h at 37°C in anaerobic conditions, and CFU quantified.

In separate experiments, a variety of soluble dietary plant fibres, including NSP isolated from dicots such as apple, runner bean, blueberry, broccoli, celery, pear, bell pepper, strawberry and tomato, were compared to NSP from the non-commelinoid monocot leek, and the commelinoid monocots oat and plantain. Each NSP was screened at 5 mg/ml for ability to block *C. difficile* interactions with Caco-2 cell monolayers.

Additional experiments were also performed to determine whether action of plantain NSP to block *C. difficile* adhesion was *via* an interaction with the intestinal epithelial monolayer or through direct interaction with bacteria. To test the former, plantain NSP was added to Caco-2 cells 30 min prior to infection as described above, but then removed by three washes with sterile PBS (1 min each; at 37°C). Monolayers were then provided with fresh antibiotic-free DMEM, infected and levels of *C. difficile* adherent to cells assessed. To test for direct interaction with bacteria, plantain NSP was incubated with *C. difficile* for 30 min, followed by centrifugation, resuspension of bacteria in antibiotic-free media and inoculation of epithelial monolayers. To assess whether the inhibitory activity of soluble NSP from plantain to block *C. difficile*-host intestinal epithelium interactions lay within an acidic (pectin-rich) or neutral polysaccharide component, Caco-2 monolayers were pre-treated for 30 min with 5 mg/ml purified, desalted Q-Sepharose anion-exchange NSP fractions, as previously described (Parsons et al., 2014).

Alternative approaches were also used to assess the impact of plantain NSP on *C. difficile* adhesion to Caco-2 cells, including Giemsa staining coupled with light microscopy (as per (Roberts et al., 2013)) and flow cytometry (based on the method of Drudy and colleagues (Drudy et al., 2001). Briefly, *C. difficile* 080042 was cultured anaerobically in 50 ml Fastidious Anaerobe Broth (Lab M; Bury, United Kingdom) without shaking for 36 h, at 37°C. Bacteria were washed and resuspended in sterile PBS, pH 7.3. For fluorochrome labelling, 1 × 10^8^ bacteria/ml were incubated with 1 μM of 2′,7′-bis-(2-carboxyethyl)-5-(and-6)-carboxyfluorescein, acetoxymethyl ester (BCECF/AM) in the dark, at 37°C for 1 h. Excess fluorochrome was removed by 5 sequential washes in sterile PBS followed by centrifugation, each for 5 min. Bacteria were re-suspended in PBS containing plantain NSP, or PBS alone and incubated for 30 min in the dark, then added to 1 × 10^6^ Caco-2 cells in single cell suspension, at 37°C for 1 h. After incubation, cells were washed three times in PBS (300 x *g*, for 20 min) to remove any non-adherent bacteria. After the final wash, pellets were resuspended in 1 ml PBS and subjected to flow cytometry using a FACScan flow cytometer (Becton, Dickinson Ltd.; Wokingham, United Kingdom). A total of 10,000 events were acquired, with data analysed using Cell Quest software (Becton Dickinson). Mean fluorescence intensity (MFI) using the geometric mean of each sample was used to assess bacterial adherence to Caco-2 cells.

### 2.7 Epithelial Cell Chloride Channel Activation Assay

Cellular chloride secretion was assessed using a colorimetric assay that measures cellular iodide efflux as a surrogate for monitoring chloride ion channel activity, as per Tang and Wildey (2004). Caco-2 cells were seeded in 96-well plates at 2.5 × 10^4^ cells/well and incubated for 24 h at 37°C. Cells were then loaded with 100 µl pre-warmed iodine loading buffer and incubated for 4 h at 37°C. Following three washes with sterile PBS, and incubated for 30 min with plantain NSP (2.5–10 mg/ml) or chloride ion channel agonists, forskolin (200 μM; Sigma) or RP107 (100 μM; Sigma) as a positive controls (Noel et al., 2006). Following incubation, cells were lysed with 1% (v/v) Triton X-100 in deionised water. Intracellular iodine concentration was determined using the modified Sandell-Kolthoff reaction, and absorbances measured at OD_410nm_ (Tang and Wildey, 2004). Chloride channel blockers, cystic fibrosis transmembrane conductance regulator (CFTR) inhibitor Inh-172 (12.5–200 μM; Sigma) (Linsdell, 2014), or 5-nitro-2-(3-phenylpropylamino)benzoic acid (NPPB, 50–800 μM; Sigma) (Keeling et al., 1991), were evaluated for their ability to block cellular chloride channel activation, and were present during the 4 h iodine-loading step prior to incubation with soluble NSP from plantain, RP107 or forskolin.

### 2.8 Treatment of Intestinal Epithelial Cells and Organoids With *Clostridioides difficile* Toxins

Purified *C. difficile* TcdA and TcdB, isolated from the highly toxic strain reference strain VPI10463, were provided by Dr Clifford Shone (Public Health England; Porton Down, United Kingdom) (Maynard-Smith et al., 2014). Binary toxin (CDT) was generated from combination (1:2) of purified component CDTa and activated component CDTb (precursor CDTb digested with trypsin), produced and purified from a ribotype 027 strain, by Drs Panagiotis Papatheodorou and Carsten Schwan (Institute for Experimental and Clinical Pharmacology and Toxicology, Albert-Ludwigs University Freiburg, Germany) (Schwan et al., 2009; Aktories et al., 2018).

Serial dilution of each toxin was made in sterile culture media to achieve final test concentrations between 1–1,000 ng/ml. Caco-2 cells were seeded at 2 × 10^5^ cells/well in 6-well tissue culture plates and incubated for 24 h. Following three washes with sterile PBS, monolayers were pre-treatment for 30 min with or without 10 mg/ml soluble NSP from plantain. Cells were then treated with toxins for up to 24 h.

Intestinal organoids, on day 4 of culture, were pre-treated with 10 mg/ml plantain NSP or saline vehicle, followed by treatment with *C. difficile* toxins (1–100 ng/ml). Images were taken using an Axiovert 25 microscope (Zeiss) and a 20X objective lens with a Hitachi HV-C20A camera before treatment and 24 h post-toxin treatment. Organoid circularity (as a measure of cytotoxicity) was scored as previously described (Jones et al., 2019).

### 2.9 Immunoblot Assessment of Toxin-Mediated Mono-Glucosylation of the Intracellular GTP-Binding Protein, Rac1

*C. difficile* toxin mediated effects were assessed by immunoblot using two monoclonal antibodies raised against Ras-related C3 botulinum toxin substrate (Rac1); clone 102 (BD Transduction Lab; Oxford, United Kingdom) previously shown to lose affinity for Rac1 following *C. difficile* toxin-mediated mono-glucosylation (Gerhard et al., 2008) and clone 238a (Abcam; Cambridge, United Kingdom) which recognises both non-glucosylated and mono-glucosylated Rac1 isoforms (Gerhard et al., 2008). Cells were lysed in ice cold 20 mM Tris-HCl buffer, pH 7.4. Whole-cell protein lysates (25 µg) were separated using 4–20% acrylamide gradient gels on SDS-PAGE at 150 V for 2 h, followed by electro-transfer to nitrocellulose membrane (100 V, for 1 h). Nitrocellulose membranes were blocked using 1% (v/v) bovine serum albumin (BSA) in PBS containing 0.1% (v/v) Tween-20 overnight at 4°C, then incubated with primary antibody (1:1,000) for 2 h at room temperature, followed by three 5 min washes with PBS-0.1% (v/v) Tween 20. Blots were incubated with horse radish peroxidase (HRP)-conjugated rabbit anti-mouse Immunoglobulin antibody (1:2,000) for 1 h at room temperature. Membranes were again washed three times with PBS-0.1% (v/v) Tween 20 and protein bands visualised with SuperSignal West Dura Extended Duration Substrate (Thermo-Fisher Scientific; Loughborough, United Kingdom) and quantified using Image Lab Software v3.0.1 (Biorad; Hemel Hempstead, United Kingdom).

### 2.10 Quantification of Inflammatory Cytokine Release

Harvested media from human cell-lines was assayed for the presence of pro-inflammatory IL-8 using a solid-phase sandwich enzyme-linked immunosorbent assay (ELISA) (Human Elipair Kit; Abcam, Cambridge, United Kingdom). For murine intestinal organoid cultures, media was assayed for the CXCL1/KC chemokine using a Duoset mouse CXCL1/KC ELISA kit (R & D systems, Abingdon, United Kingdom). For both assays, capture antibody in 0.5 M carbonate-bicarbonate buffer (pH 9.6) was coated onto a 96-well high-binding γ-irradiated ELISA plates (Costar) overnight at 4°C. Following three washes with PBS-0.1% (v/v) Tween 20, plates were blocked with 1% (w/v) bovine serum albumin for 2 h at room temperature. Triplicate treatment samples (100 µl) of cell-free medium were then incubated overnight at 4°C, plates washed three times with 12.0 µl wash buffer, and the respective biotinylated detection antibody added for 2 h at room temperature. Subsequently, ExtrAvidin peroxidase (Sigma; Poole, United Kingdom) was added to each well, incubated for 20 min at room temperature. Plates were again washed three times with PBS-Tween, developed with SigmaFast *o*-phenylenediamine substrate (Sigma) for 10 min and colour development stopped with 100 µl 4 M H_2_SO_4_. ELISAs were measured on a Tecan Sunrise plate reader (Tecan; Reading, United Kingdom) at OD_492nm_, with a reference filter set at OD_750nm_. Treatment samples were quantified against calibration curves of recombinant human IL-8 (0–2000 pg/ml) and mouse KC (0–1,000 pg/ml) standards in culture medium.

### 2.11 Assessment of Cellular Apoptosis

Cells were seeded at 1 × 10^4^ cells/ml in clear bottomed white wall 96-well tissue culture plates (Corning/Costar) and cultured overnight at 37°C. Following treatment with purified toxins and/or *C. difficile* clinical isolates, cell monolayers were assayed using a commercial Caspase-Glo 3/7 assay (Promega; Southampton, United Kingdom), as per manufacturer instructions. Luminescence was detected using the Infinite F200 plate reader (Tecan). Experiments were performed in triplicate, with background luminescence determined from wells containing culture medium alone.

### 2.12 Assessment of Cellular Cytotoxicity

Epithelial cell viability during NSP treatments and in response to *C. difficile* infection or toxin treatment was monitored by measurement of adenylate kinase (normally found within intact cells) released to the culture medium from Caco-2 cells using a ToxiLight bioassay (Lonza; Slough, United Kingdom). Intestinal organoid cytotoxicity was measured as previously described (Jones et al., 2019).

### 2.13 Statistical Analysis

Data are expressed as mean ± SD, unless otherwise stated. N numbers indicate the total number of independent experiments performed, where each experiment was performed with at least *n* = 3 replicates for any individual treatment group, unless otherwise indicated. Independent sample groups were first assessed for normality (Shapiro-Wilks test) and equality of variances (Levene’s test). Two-sample comparisons were performed using unpaired t-test or non-parametric Mann-Whitney U test as appropriate. Multiple treatment groups were analysed using either parametric one-way analysis of variance (ANOVA) or non-parametric Kruskal-Wallis test (Stats-Direct v3.0.171; Sale, United Kingdom). Differences were considered significant when *p* < 0.05.

## 3 Results

### 3.1 Soluble NSP From Plantain Inhibits Intestinal Epithelial Adherence of *Clostridioides difficile* Clinical Isolates, Irrespective of Toxin Status and Ribotype

**FIGURE 1:**
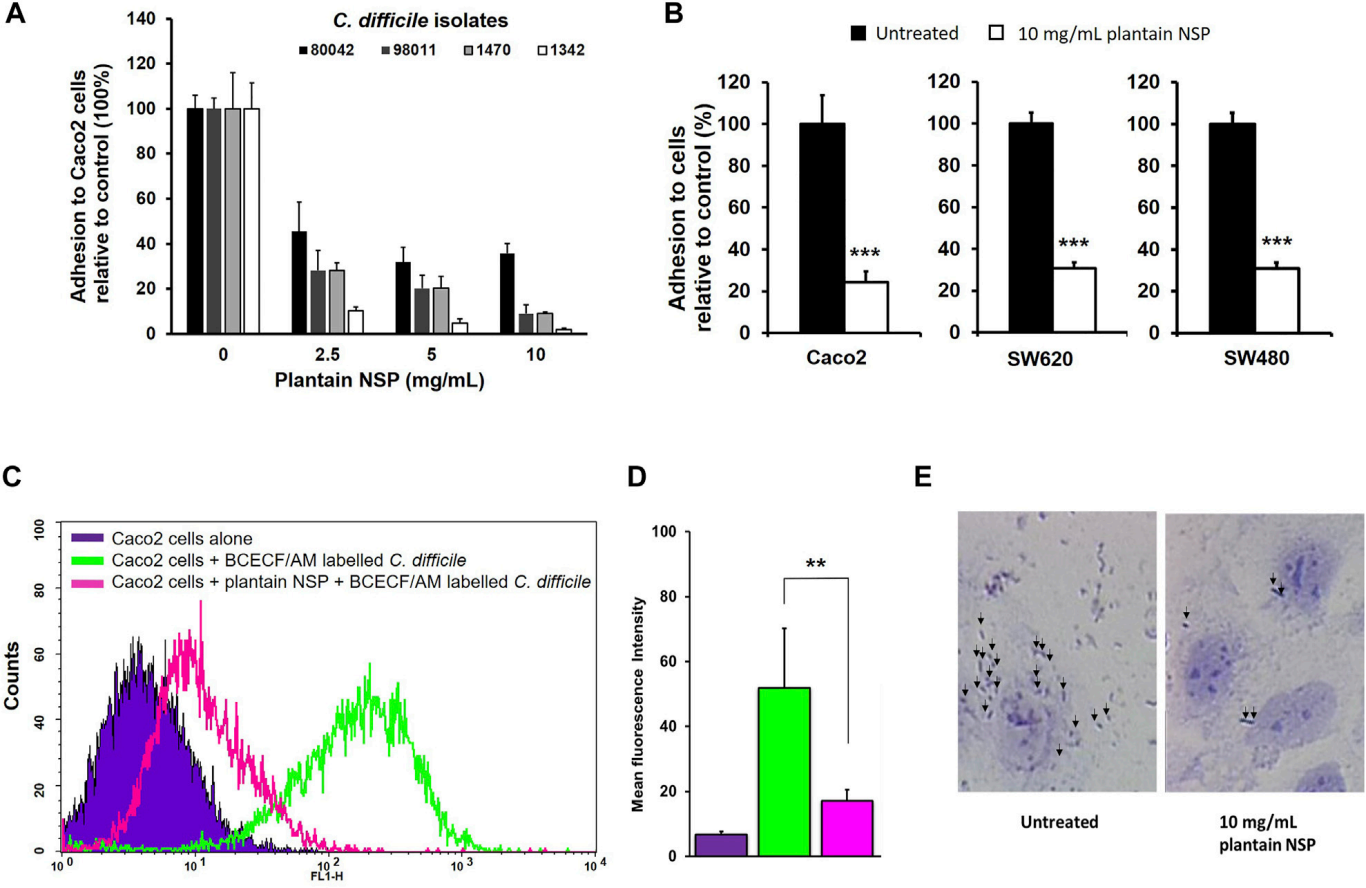
Soluble non-starch polysaccharides from plantain inhibits *C. difficile* adhesion to intestinal epithelial cells in a dose-dependent manner. **(A)** Adhesion of *C. difficile* isolate 80042 to confluent Caco-2 [Caco2] monolayers are significantly inhibited in the presence of plantain (NSP). **(B)** Pre-treatment with 10 mg/ml plantain NSP, blocked adhesion of C. *difficile* 80042 to three different intestinal cell-lines Caco-2, SW480 and SW620. Adhesion is expressed relative to CFU/mL found in the absence of plantain soluble dietary fibre, set as 100% (*N* = 3 experiments, *n* = 3 replicates; **p < 0.01, ***p < 0.001; Kruskal-Wallis test or Mann Whitney U test). **(C)** Flow cytometric analysis of 1 × 10^6^ Caco-2 cells in suspension, either uninfected (blue), co-incubated with 1 × 10^8^ BCECF/AM-labelled *C difficile* 80042 for 1 h, either without (green), or with 30 min pre-treatment with 10 mg/ml plantain NSP (pink). **(D)** Data are based on 10,000 events, and representative of *N* = 4 separate experiments. Significant effect of NSP, as assessed by shift in mean fluorescence intensity, compared to infected controls (**p < 0.01, Kruskal-Wallis test). **(E)** Giemsa-stained *C. difficile* 80042 inoculated Caco-2 cells in the absence and presence of 10 mg/ml plantain NSP. Black arrows indicate adherent bacteria.

Soluble NSP from plantain was observed to dose-dependently block adhesion of clinical *C. difficile* isolates to Caco-2 intestinal epithelial cell monolayers. Peak inhibition of *C. difficile* adhesion to Caco-2 cells was seen following pre-treatment with plantain NSP at 10 mg/ml for ribotype 027, toxin positive isolate *C. difficile* 80042 (64.2 ± 4.4% inhibition) was seen compared to untreated controls (*N* = 3, n = 3, *p* < 0.001, Kruskal-Wallis test), see Figure 1A. Plantain NSP also dose dependently reduced *C. difficile* adhesion of other *C. difficile* isolates, including the ATCC reference strain *C. difficile* 1470 to Caco-2 cells (peak inhibition seen at 10 mg/ml, 81.2 ± 2.4% in comparison to the untreated control (*N* = 3, *n* = 3, *p* < 0.001); Figure 1A. Using the optimum inhibitory dose of 10 mg/ml plantain NSP, similar levels of blockade of *C. difficile* 80042 interaction to epithelial cells was observed using two other colonic cell-lines SW620 (69.2 ± 3.0% inhibition) and SW480 (70.6 ± 1.4%) as was seen using Caco-2 cells (75.6 ± 5.1%), compared to untreated controls (*N* = 3; *p* < 0.001, Mann-Whitney U test), Figure 1B. Alternative approaches to measure bacterial adhesion, by flow cytometry (Figures 1C,D) and Giemsa staining coupled with light microscopy (Figure 1E), also revealed marked reduction in adhesion of *C. difficile* to Caco-2 cells in the presence of plantain NSP.

**FIGURE 2:**
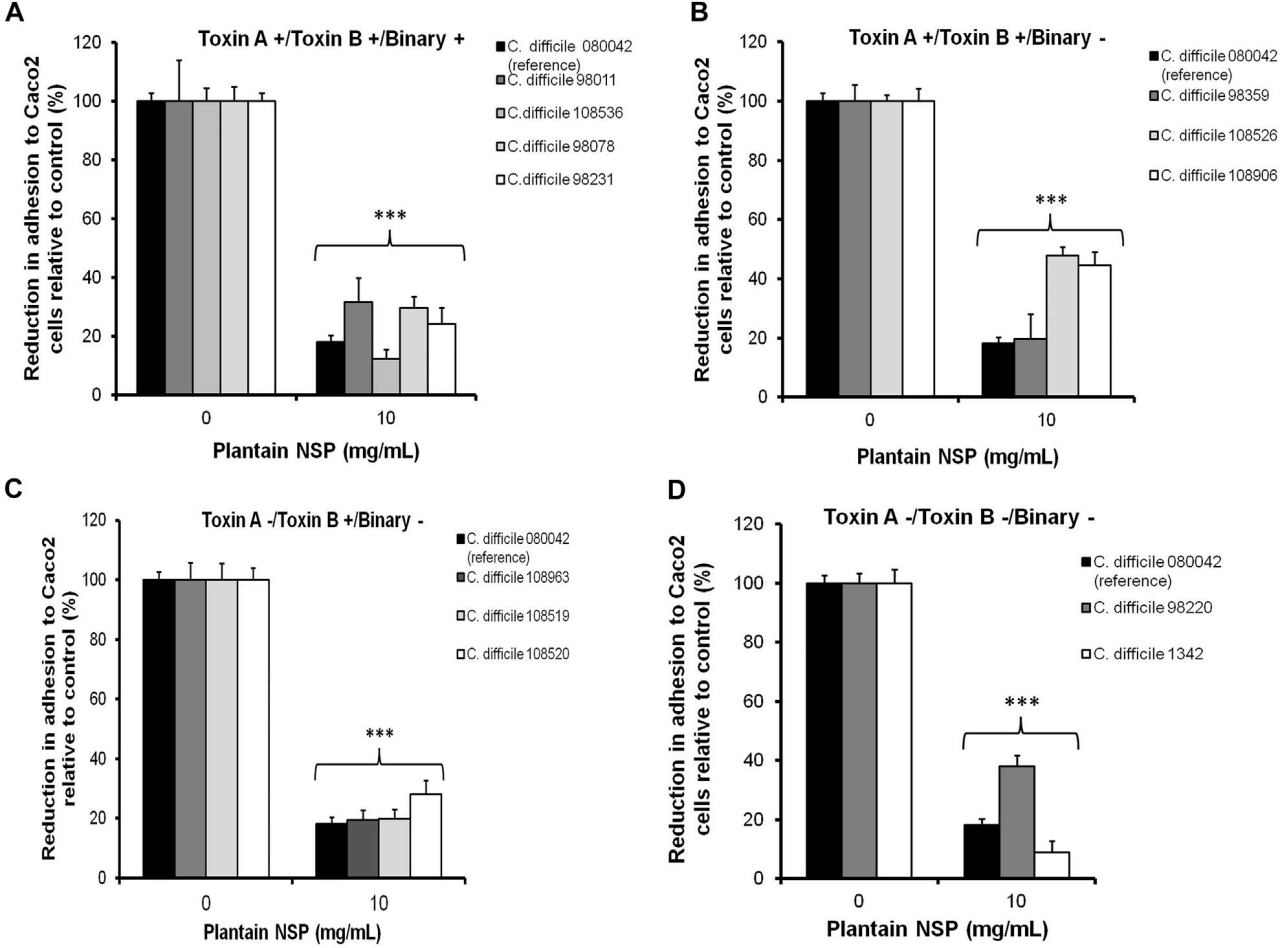
Soluble NSP from plantain inhibits the epithelial adhesion of a panel of *C. difficile* clinical isolates differing in toxin expression and ribotype. Caco-2 [Caco2] cells underwent pre-treatment with 10 mg/ml plantain NSP for 30 min prior to inoculation with *C. difficile* at a multiplicity of infection (MOI) of 100. Plantain NSP significantly inhibited adhesion to Caco-2 cell monolayers, of *C. difficile* clinical isolates positive for **(A)** all toxins (A, B and binary), **(B)** positive for toxin A and toxin B, **(C)** Toxin B only, and **(D)** toxin null strains. Clinical laboratory *C. difficile* isolate 80042 (ribotype 027) was used as a positive reference for plantain NSP inhibition of bacteria-epithelial cell adhesion, with adhesion to Caco-2 cells expressed relative to CFU/mL found in each untreated control (set as 100%). Significant differences from untreated control, ***p < 0.001, *N* = 3 experiments, *n* = 3 replicates; Kruskal-Wallis test).

Plantain NSP (at 10 mg/ml) also significantly reduced adhesion of a panel of *C. difficile* clinical isolates differing in their geographical origin, toxin expression and PCR ribotype (Table 1) to Caco-2 cells, with an overall median reduction for all isolates tested of 73.8% (range 52.2–98.6%) in comparison to the untreated inoculated monolayers (Figures 2A–D, for all; *p* < 0.001; Kruskal-Wallis test). These results demonstrate that the inhibitory effect of plantain NSP is independent of *C. difficile* toxin expression and ribotype status.

### 3.2 Soluble NSP From Other Plant Sources can Inhibit the Epithelial Adhesion of *Clostridioides difficile*, but Not as Effectively as NSP From Plantain

**FIGURE 3:**
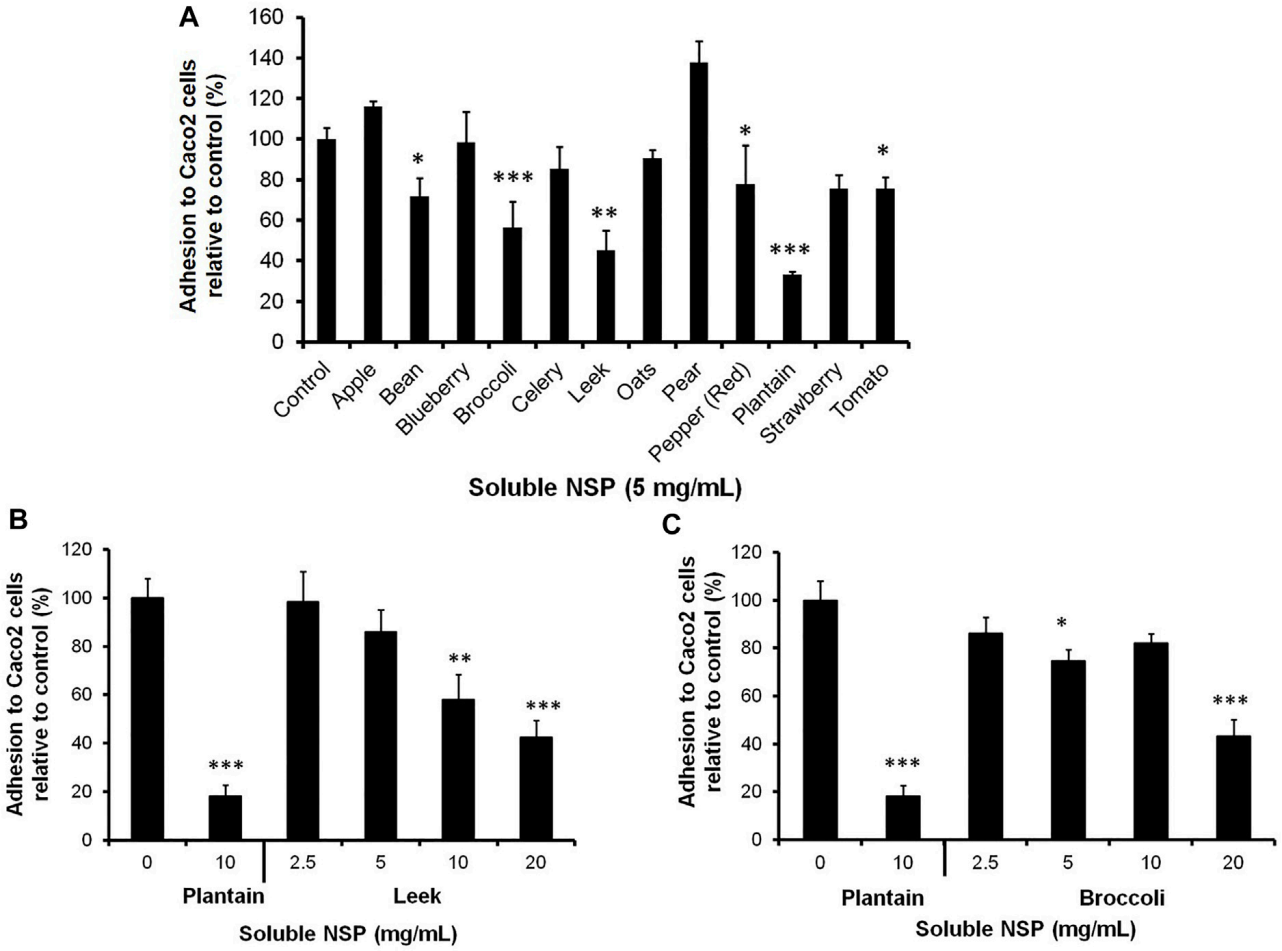
Evaluation of a range of dietary plant soluble non-starch polysaccharides (NSP) for their ability to prevent the adhesion of *C. difficile* to intestinal epithelial cells *in vitro*. **(A)** Adhesion of *C. difficile* isolate 080042 to confluent Caco-2 [Caco2] cells monolayers is significantly inhibited in the presence of 5 mg/ml NSP from runner bean, broccoli, leek, bell pepper, plantain and tomato. Further analysis showed *C. difficile* epithelial adhesion to be reduced in a dose dependent manner by, **(B)** leek NSP and **(C)** broccoli NSP, compared to blockade seen with 10 mg/ml plantain NSP. Adhesion was measured as relative to CFU/mL quantified in the absence of NSP (set as 100%). All *N* = 3 experiments, *n* = 3–4; excepting for leek NSP (*N* = 1, *n* = 3); *p < 0.05, **p < 0.01 and ***p < 0.001; Kruskal-Wallis test).

To screen for inhibitory activity, Caco-2 cell monolayers underwent pre-treatment using soluble NSP from a range of dietary plants prior to infection with *C. difficile* 80042 (Figure 3A). Pre-incubation of intestinal cells with 5 mg/ml NSP from broccoli, leek and plantain resulted in the highest levels of inhibition of *C. difficile* 80042 adhesion to Caco-2 cells (>43% inhibition; *p* ≤ 0.01 (*N* = 3, *n* = 3–4 for broccoli NSP and plantain NSP; *N* = 1, *n* = 3 for leek NSP). NSP from runner bean, bell pepper or tomato was observed to be less effective (>22% inhibition; *p* < 0.05) whilst others such as apple, blueberry, celery, oat, pear and strawberry, had little or no inhibitory effect (*N* = 3, *n* = 3–4); see Figure 3A. Further study of leek NSP and broccoli NSP indicated a dose-dependent inhibitory effect on *C. difficile*-epithelial cell adhesion, with peak inhibition seen at 20 mg/ml (leek NSP, 57.5 ± 6.5%; and broccoli NSP*,* 56.9 ± 7.2% compared to untreated controls (100%); both *p* < 0.001). Both these dietary plant soluble fibres had a lower efficacy than that seen with plantain NSP; used as a positive control at 10 mg/ml, where the epithelial adhesion of *C. difficile* was reduced by 81.8 ± 4.6% (*p* < 0.001; *N* = 3, *n* = 3); see Figures 3B,C.

### 3.3 Soluble NSP From Plantain Inhibits the Epithelial Adherence of *Clostridioides difficile via* an Effect on the Intestinal Epithelium

Plantain NSP appears to exert its inhibitory effect on *C. difficile* adhesion *via* action on the epithelium. When plantain NSP (10 mg/ml) was added to Caco-2 cell monolayers for 30 min, then removed by three washes with sterile PBS, levels of adherent *C. difficile* were observed to be significantly reduced (59.2 ± 5.0%) compared to the untreated inoculated controls (*p* < 0.01; Kruskal-Wallis test), albeit lower than that seen in experiments where plantain NSP was added to cells for 30 min without removal before infection (92.9 ± 2.0%, compared to the untreated control; *p* < 0.001; Kruskal-Wallis test). In contrast, pre-incubation of plantain NSP directly with bacteria for 30 min, followed by removal of soluble dietary fibre from bacteria by centrifugation, resulted in no significant inhibition of *C. difficile* adhesion compared with untreated controls (see Supplementary Figure S2).

In addition, treatment of Caco-2 cells with 2.5–20 mg/ml plantain NSP also resulted in a dose-dependent increase in cellular chloride ion channel activity, with peak activity at 20 mg/ml plantain NSP (5.4 ± 0.4-fold increase in comparison to the untreated control; *p* < 0.001 Kruskal-Wallis test, *N* = 3, n ≥ 3). This level of activation was similar to that seen with chloride ion channel agonists forskolin (200 μM) and RP107 (100 μM), which increased chloride ion channel activity by 5.4 ± 0.5 and 8.2 ± 1.0-fold respectively, in comparison to the untreated vehicle controls (*p* < 0.001). Other dietary plant NSP’s (at 5 mg/ml), previously shown to have little or no inhibitory action on *C. difficile* adhesion to Caco-2 cells, exhibited less efficacy with oat and runner bean NSP increasing chloride ion channel activity at a much lower levels (2.2 ± 0.6 and 2.0 ± 0.3 fold, respectively; both *p* < 0.001), with tomato, pear, bell pepper and apple NSP actually reducing chloride ion channel activity in comparison to the untreated controls (see Supplementary Figure S3). Chloride ion channel activity in Caco-2 cells induced by 10 mg/ml plantain NSP, was significantly inhibited by inhibitors NPPB and Inh-172 in dose-dependent manner; with peak levels of inhibition seen using 800 µM NPPB and 200 µM Inh-172, reducing chloride efflux by 94.7 ± 10.0% and 86.9 ± 8.5% respectively, in comparison to the vehicle-treated controls (both *p* < 0.001). However, bacterial interaction assays indicated that in the presence of NPPB or Inh-172, pre-treatment of Caco-2 cells with 10 mg/ml plantain NSP still possessed significant ability to reduce *C. difficile* 80042 adhesion. *C. difficile* adhesion to Caco-2 cells was significantly decreased when treated with chloride ion channel agonist.

RP107, with an observed reduction in adhesion of 23.7 ± 9.0% (at 100 μM), from vehicle treated controls (set at 100%) (*p* < 0.01; Kruskal-Wallis test), with this action reversed in the presence of chloride ion channel antagonists NPPB or Inh-172, increasing bacterial adhesion by 7.9 ± 8.1% and 4.5 ± 4.1% respectively above the level seen in controls (100%); see Supplementary Figure S3).

### 3.4 The Inhibitory Effect of Plantain NSP on *Clostridioides difficile* Intestinal Epithelial Cell Adherence is Mediated Primarily by the Acidic (Pectin-Rich) Polysaccharide Fraction

**FIGURE 4:**
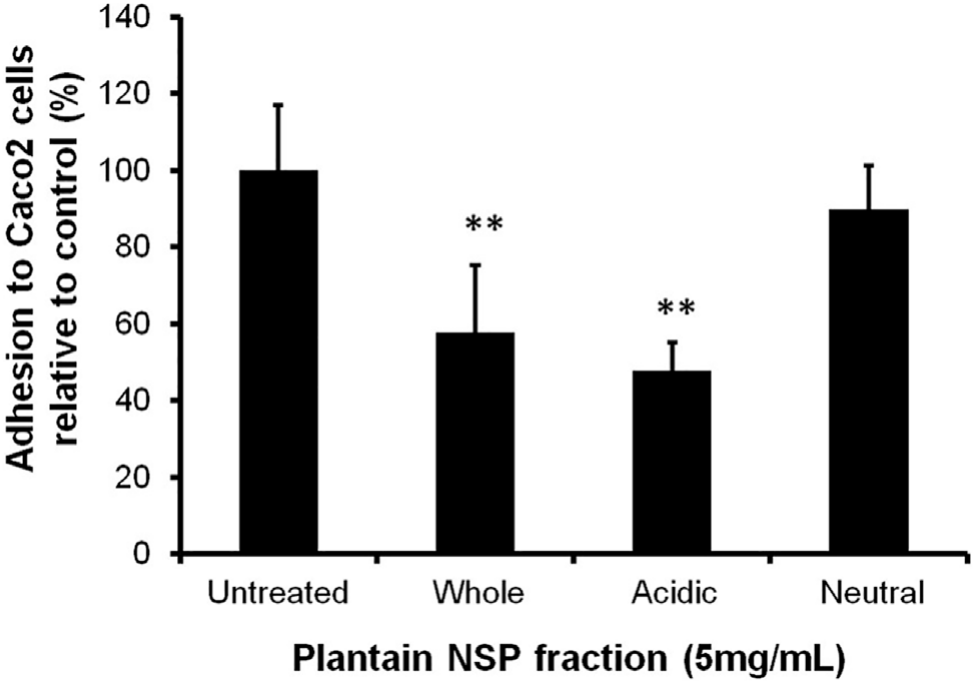
The inhibitory activity of soluble NSP from plantain to block *C. difficile*-host intestinal epithelium interaction lies within an acidic (pectin-rich) polysaccharide component. At 5 mg/ml, the Q-Sepharose purified acidic polysaccharide fraction of plantain NSP significantly blocked adhesion of *C. difficile* to confluent Caco-2 [Caco2] cell monolayers, whereas the neutral fraction had no significant effect compared to the untreated control (*N* = 3 experiments, *n* = 3 replicates, **p < 0.01, Kruskal-Wallis test).

At 5 mg/ml, the acidic (pectin-rich) polysaccharide fraction of plantain NSP, significantly blocked adhesion of *C. difficile* isolate 080042 to intestinal Caco-2 cell-line by 52.3 ± 7.2% (*p* < 0.01), and was at least as effective as the unfractionated plantain soluble fibre, which reduced adhesion by 62.3 ± 17.1% (*p* < 0.01). Conversely, pre-treatment of Caco-2 cells with 5 mg/ml neutral polysaccharide fraction (unbound to Q-Sepharose) of plantain NSP showed significantly reduced ability to inhibit *C. difficile* epithelial adhesion (Figure 4).

### 3.5 Soluble NSP From Plantain Inhibits the Epithelial Adherence of *Clostridioides difficile* Spores

Soluble NSP from plantain, at 10 mg/ml, significantly reduced the adhesion of all five *C. difficile* isolate spore preparations to Caco-2 cells, with inhibition ranging from 27.9 ± 5.4% to 33.8 ± 6.9% (all *p* ≤ 0.05, Kruskal-Wallis test); see Table 2. Blockade of spores was less than that observed with the corresponding vegetative *C. difficile* bacterium, from which the spores were purified, at the same MOI 100, where pre-incubation with 10 mg/ml plantain NSP resulted in inhibition ranging from 78.9 ± 3.1% to 97.9 ± 1.5% (for all, *p* < 0.001). It is worth noting that *C. difficile* spores (from all isolates) showed markedly greater adhesion to Caco-2 cells (85 ± 7.3% versus 4.0 ± 0.6% adhesion of the original inoculum of spores and vegetative cells (1 × 10^7^ CFU/ml), respectively; *N* = 5 isolates).

**TABLE 2:** Soluble NSP from plantain inhibits epithelial adhesion of *C. difficile* spores and vegetative cells.

*C. difficile* strain	% reduction in spore adhesion with plantain NSP^a^	% reduction in *C. difficile* adhesion with plantain NSP^a^
98011	30.3 ± 5.2*	93.4 ± 1.9***
108536	33.8 ± 6.9*	92.6 ± 4.3***
108526	27.9 ± 5.4**	86.7 ± 2.0***
98220	29.9 ± 8.9**	78.9 ± 3.1***
1342	32.8 ± 14.9**	97.9 ± 1.5***

a Reduction in adhesion of purified *C. difficile* isolate spores and vegetative cells to Caco-2 cells expressed as a percentage of untreated controls, set as 100%. Caco-2 cells were pre-treated with 10 mg/ml plantain non-starch polysaccharides (NSP) for 30 min before inoculation (1 × 10^7^ CFU/ml; multiplicity of infection, MOI 100). Significant differences from untreated controls, *p < 0.05, **p < 0.01 and ***p < 0.001 (data expressed as mean ± SEM for *N* = 2–4 experiments, *n* = 3 replicates; Kruskal-Wallis test).

### 3.5 Soluble NSP From Plantain Reduces *Clostridioides difficile* Toxin A/B-Induced Inflammation and Cytotoxicity, But Does Not Block Toxin-Mediated Rac1 Mono-Glucosylation Within Intestinal Epithelial Cells

#### 3.5.1 Plantain NSP Blockade of *Clostridioides difficile* Toxin A/B-Induced Inflammation

**FIGURE 5:**
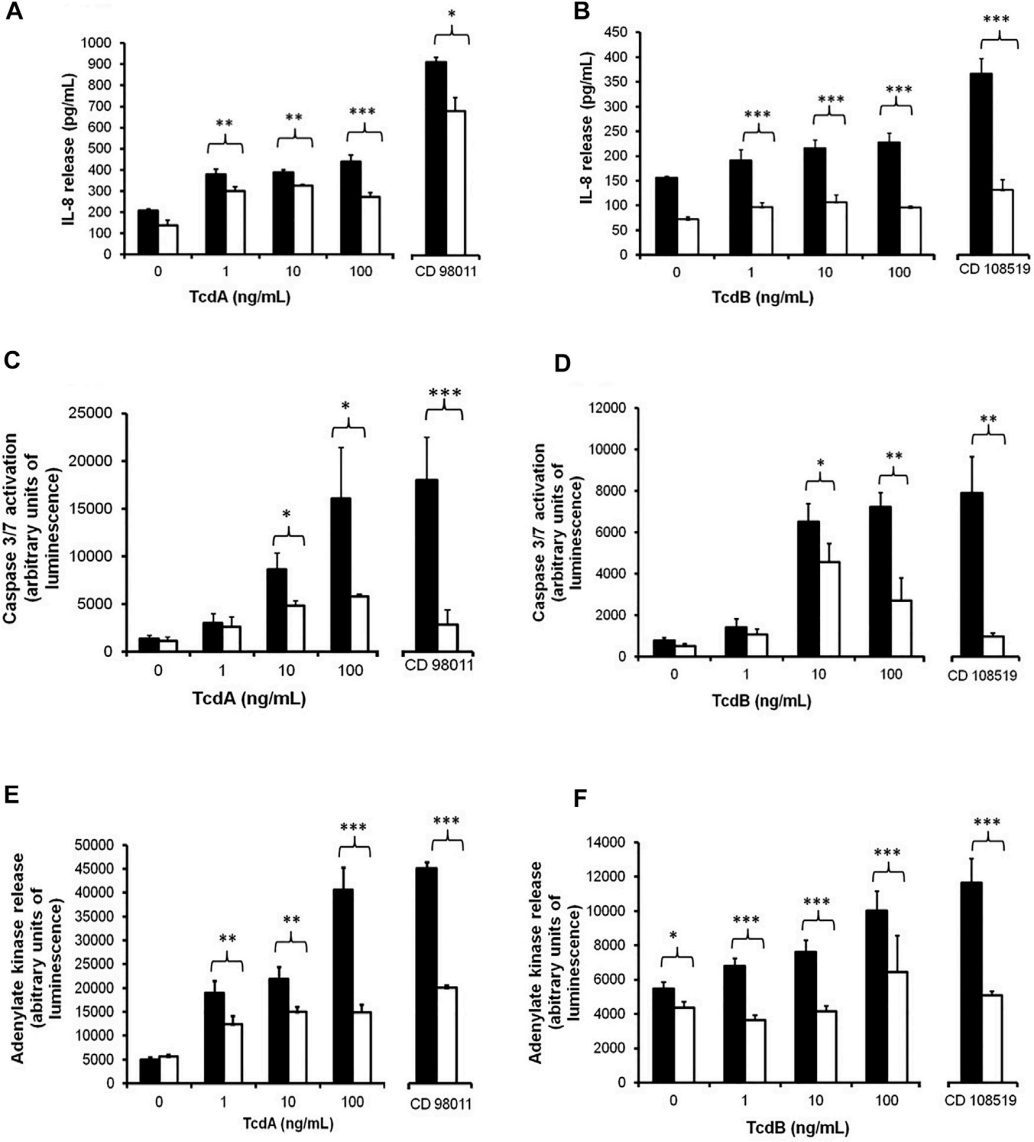
Soluble NSP from plantain reduces *C. difficile* bacterium and toxin A and toxin B mediated inflammation, cellular apoptosis and cytotoxicity in intestinal epithelial cells. Caco-2 cell monolayers were treated with *C. difficile* toxins A (TcdA) or toxin B (TcdB), at 1–100 ng/ml for 24 h, in the absence (black bars) or presence of 10 mg/ml plantain NSP (white bars). In parallel, Caco-2 cells were infected with the TcdA+/TcdB+/CDT + *C. difficile* isolate 98011 or the TcdB + *C. difficile* isolate 108519, at a multiplicity of infection (MOI) of 100 for 4 h, again in the absence or presence of plantain NSP. Media harvested from cells was analysed for release of IL-8 (by ELISA) and cytotoxicity marker adenylate kinase (by Toxilight bioassay). Apoptotic cell death was measured using the Caspase Glo 3/7 assay. In the presence of plantain NSP, TcdA- and TcdB-mediated **(A,B)** IL-8 release, **(C,D)** caspase 3/7 activation, and **(E,F)** cytotoxicity (intracellular adenylate kinase release) were all significantly reduced (*N* = 2, *n* = 3; *p < 0.05, **p < 0.01, ***p < 0.001; Kruskal-Wallis test).

*C. difficile* toxins A (TcdA) and B (TcdB) both elicited dose-dependent increases in release of pro-inflammatory cytokine IL-8 from Caco-2 cells, which were seen to be significantly blocked by 30 min pre-treatment with 10 mg/ml plantain NSP. The peak induced response seen with 100 ng/ml TcdA (445.1 ± 30.9 pg/ml IL-8 released) was reduced by 10 mg/ml plantain NSP to 270.9 ± 21.1 pg/ml (*p* < 0.001; *N* = 2, *n* = 3; Kruskal-Wallis). Treatment of Caco-2 cells with *C. difficile* 98011 (TcdA^+^, TcdB^+^ and CDT^+^) at MOI 100 for 4 h, IL-8 release increased from basal levels of 206.7 ± 7.8 pg/ml to 915.5 ± 20.6 pg/ml, and in the presence of 10 mg/ml plantain NSP, *C. difficile* mediated IL-8 release was decreased to 683.6 ± 63.7 pg/ml (*p* < 0.05); see Figure 5A. Similarly, 100 ng/ml TcdB-induced IL-8 release was also seen to be blocked by plantain NSP from 228.2 ± 18.5 pg/ml to 96.0 ± 3.3 pg/ml (both *p* < 0.001; *N* = 2, *n* = 3; Kruskal-Wallis test). Of note, the IL-8 response seen 4 h post-infection with the *C. difficile* 108519 (expressing TcdB only) was lower (at only 366.5 ± 31.0 pg/ml) than that seen with *C. difficile* 98011 expressing all three toxins (*p* < 0.001). Pre-treatment with 10 mg/ml plantain NSP also decreased *C. difficile* 108519 (TcdB^+^ only) mediated IL-8 release from Caco-2 cells (2.8-fold decrease; *p* < 0.001); Figure 5B. It was also noted that Caco-2 cell monolayers that underwent pre-treatment with 10 mg/ml plantain NSP but received no subsequent treatment with *C. difficile* toxins nor bacteria, showed marked decreases in basal secreted levels of IL-8; Figures 5A,B.

#### 3.5.2 Plantain NSP Blockade of *Clostridioides difficile* and Toxin-Induced Cytotoxicity

*C. difficile* bacterium and toxin-mediated apoptosis (caspase-3/7 activation) was also monitored in the absence and presence of plantain NSP. *C. difficile* toxins elicited cellular caspase-3 activation in a dose-dependent manner, with peak response seen at 100 ng/ml for both TcdA and TcdB (see Figures 5C,D). As per toxin-induced IL-8 release, caspase-3/7 activation was observed to be higher in Caco-2 cells that had been treated with TcdA. Pre-treatment of cells with 10 mg/ml plantain NSP resulted in a significant decrease in toxin-mediated caspase-3/7 activation (e.g. 100 ng/ml TcdA, 63.8 ± 1.6% reduction and 100 ng/ml TcdB, 58.2 ± 10.4% reduction in caspase3/7 activation (both *p* < 0.05). Following 4 h infection of Caco-2 cells with *C. difficile* 98011 (expressing all toxins) there was a 13.1 ± 3.3-fold increase in caspase-3/7 activation in comparison to the untreated controls. In the presence of 10 mg/ml plantain NSP, there was a significant reduction in *C. difficile* 98011 mediated caspase-3/7 activation (84.1 ± 7.0% reduction compared that seen in absence of plantain NSP, *p* < 0.001; *N* = 2, *n* = 3; Kruskal-Wallis test); see Figure 5C. Cellular caspase-3/7 activity was also increased (10.1 ± 1.6 fold) after infection with *C. difficile* 108519 (TcdB expressing only) albeit at a lower level than that seen with *C. difficile* 98011 (Figure 5D). Again, pre-incubation with 10 mg/ml plantain NSP resulted in a marked reduction (87.6 ± 1.4%) in bacteria-induced caspase 3/7 activation (*p* < 0.001). As was seen with IL-8 response, there was a small, but notable, decrease in caspase 3/7 activation in cells pre-treated with plantain NSP alone.

As seen with other toxin-mediated effects, *C. difficile* toxins TcdA and TcdB evoked a dose-dependent increase in adenylate kinase release from Caco-2 cells, with the peak responses seen at 100 ng/ml for both toxins (Figures 5E,F). Again, adenylate kinase release was significantly higher in cells treated with TcdA (8.2 ± 0.9-fold increase) compared to TcdB (1.8 ± 0.2-fold increase); *p* < 0.001; *N* = 2, *n* = 3. Pre-incubation with 10 mg/ml plantain NSP significantly decreased adenylate kinase release in 1, 10, and 100 ng/ml TcdA- and TcdB-treated cells (all doses, *p* ≤ 0.01). As an example, pre-treatment with plantain NSP in cells treated with 100 ng/ml TcdA and TcdB lead to significant decrease in adenylate kinase release by 63.3 ± 4.1% and 35.7 ± 9.2%, respectively (both *p* < 0.001; see Figures 5E,F). Following 4 h infection of Caco-2 cells with *C. difficile* 98011, an 8.9 ± 0.3-fold increase was observed in adenylate kinase release in comparison to untreated controls, which was significantly decreased in cells pre-incubated with 10 mg/ml plantain NSP (47.3 ± 1.03% reduction; *p* < 0.001; Figure 5E). Whilst adenylate kinase release increased by 2.1 ± 0.3-fold in cells infected with *C. difficile* 108519 (expressing TcdB only), the response was lower than that seen with *C. difficile* 98011 (expressing all toxins), with pre-incubation of Caco-2 cells with 10 mg/ml plantain NSP resulting in significant reduction of cellular adenylate kinase release (56.2 ± 1.9%) in comparison to that seen in the absence of plantain NSP (*p* < 0.001; Figure 5F). In some experiments, basal levels adenylate kinase release appeared to be lower in Caco-2 cells receiving with plantain NSP alone.

**FIGURE 6:**
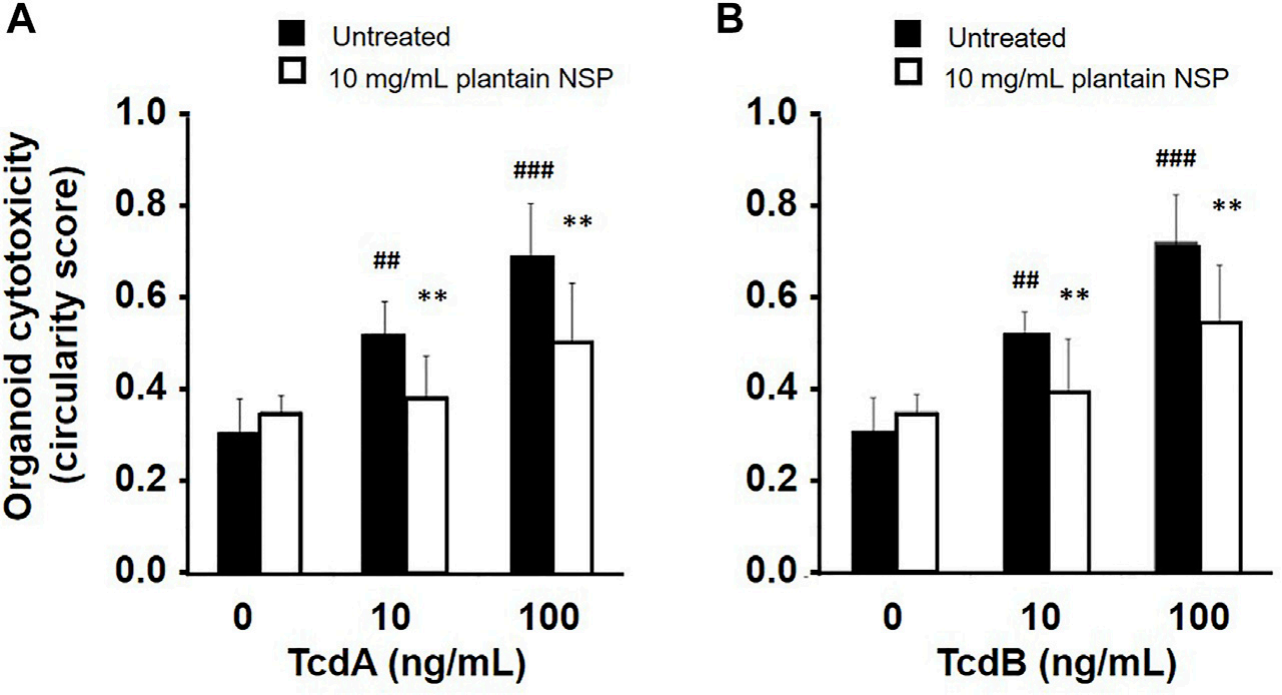
Soluble NSP from plantain inhibits *C. difficile* toxin-induced cytotoxicity in intestinal crypt stem cell-derived organoids. Intestinal organoid cultures were pre-treated for 30 min with 10 mg/ml plantain NSP before treatment with 10 and 100 ng/ml of *C. difficile* toxins, **(A)** TcdA or **(B)** TcdB. Plantain NSP significantly reduced TcdA- and TcdB-induced cytotoxicity (as assessed by rounding/circularity score) compared to vehicle (sterile PBS, pH 7.3) pre-treated controls. Organoid rounding was quantified as previously described (Parsons et al., 2015), with circularity values possible between 0 and 1, and where a score of 1 indicates a perfect circle. Data expressed as mean ± SD (*N* = 4). Significant differences of toxin-induced response to untreated controls, ^##^ *p* < 0.01, ^###^ *p* < 0.001, and plantain NSP versus vehicle pre-treated controls; **p < 0.01; One-way ANOVA, with Dunnett’s post-hoc test.

Treatment of stem cell derived 3-D intestinal organoids from C57BL/6J mice with purified *C. difficile* toxins, both TcdA and TcdB, dose-dependent increased organoid rounding (cytotoxicity) over 24 h. Morphological changes induced by 100 ng/ml TcdA (circularity score, 0.64 ± 0.05 [mean ± SD]) and TcdB (0.71 ± 0.09) were similar to that seen following treatment with 100 ng/ml TNFα (0.71 ± 0.08); compared to untreated controls (0.38 ± 0.03); *N* = 4; *p* < 0.01 one-way ANOVA, see Supplementary Figure S4. Pre-treatment of organoids with 10 mg/ml plantain NSP 30 min prior to addition of toxin, reduced the level of cytotoxicity induced by all doses of TcdA and TcdB. At 100 ng/ml, TcdA mediated circularity (0.69 ± 0.12) was reduced to 0.50 ± 0.13, and TcdB induced circularity reduced from 0.72 ± 0.10 to 0.54 ± 0.12]; *N* = 4; both *p* < 0.01 (see Figure 6). Levels of chemokine CXCL1/KC dose-dependently released to the medium from toxin-treated organoids, were also observed to be reduced by pre-treatment of cultures with 10 mg/ml plantain NSP. CXCL1/KC released from organoids treated with 100 ng/ml TcdA or TcdB (409.4 ± 31.7 pg/ml or 532.9 ± 28.1 pg/ml released to media in 24 h) were reduced with plantain NSP by 67.7 ± 4.3 pg/ml and 65.8 ± 4.8 pg/ml respectively; both *p* < 0.01 one-way ANOVA.

#### 3.5.3 Plantain NSP Does Not Block of *Clostridioides difficile* Toxin A- and Toxin B-Induced Intracellular Glucosylation of Rac1

**FIGURE 7:**
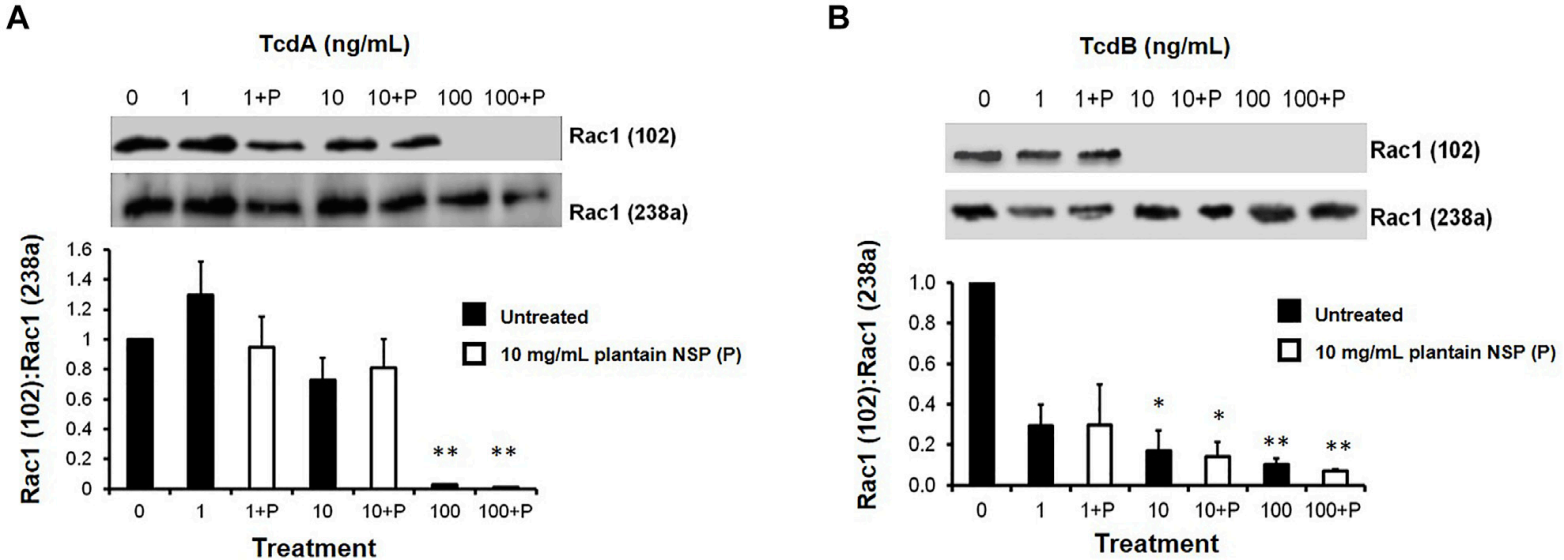
*C. difficile* toxins A and B mediate increased Rac1 glucosylation within intestinal epithelial cells, which is unaffected by pre-treatment with soluble NSP from plantain. Immunoblot analysis shows an increase in Rac1 glucosylation following treatment with 10 ng/ml **(A)** toxin A (TcdA) for 24 h and **(B)** toxin B (TcdB) for 48 h, which is unaffected by pre-treatment of Caco-2 cells with 10 mg/ml soluble plantain NSP (P). Protein bands were analysed by chemiluminescence image densitometry. Mono-glucosylated Rac1 levels (assessed with antibody 102) were normalised to total Rac1 (assessed with antibody 238a), with representative blots shown for *N* = 3 experiments, *n* = 3 replicates. Significant differences from non-toxin treated controls, *p < 0.05, **p < 0.01 and ***p < 0.001; Kruskal-Wallis test.

Treatment of Caco-2 cells with 10 ng/ml TcdA elevated cellular levels of mono-glucosylated Rac1 (as indicated by a loss of binding of antibody clone 102 to Rac1) 10 h post-toxin treatment, with peak mono-glucosylation of Rac1 observed at 24 h compared to vehicle-treated control cells; *N* = 3, *n* = 3; *p* < 0.01 (see Supplementary Figure S5). Treatment of cells with 10 ng/ml TcdB also revealed elevated cellular levels of mono-glucosylated Rac1, but this was only significant 48 h post-toxin treatment; *N* = 3, *n* = 3; *p* < 0.01 (see Supplementary Figure S5). Notably, neither TcdA nor TcdB mediated mono-glucosylation of intracellular Rac1 was seen to be blocked in Caco-2 cells pre-treated with 10 mg/ml plantain NSP (see Figure 7).

### 3.6 Pre-treatment With Plantain NSP Reduces the Pathogenic Effects of *Clostridioides difficile* Binary Toxin in Intestinal Epithelial Cells

**FIGURE 8:**
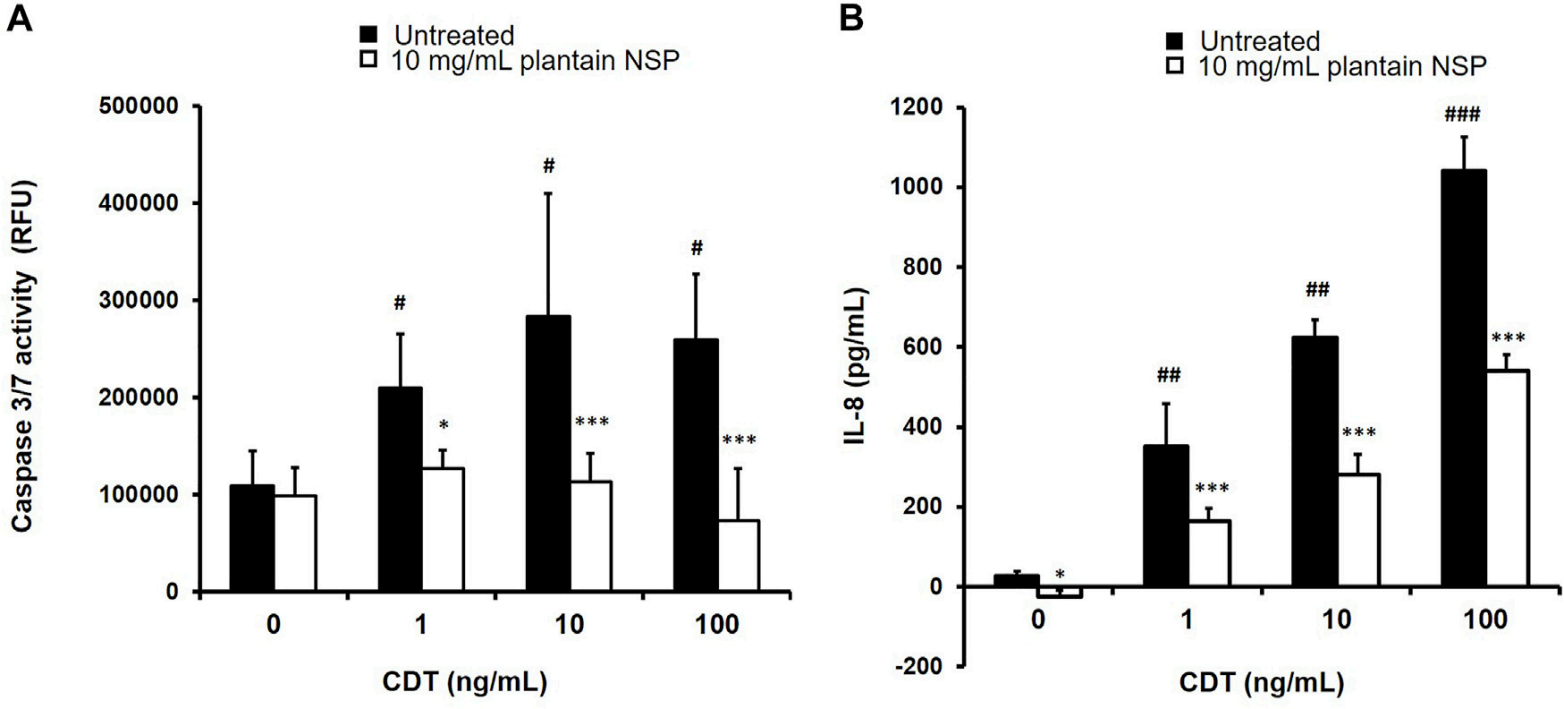
Soluble NSP from plantain counters the pathogenic actions of *C. difficile* binary toxin (CDT) on human colonocytes. Treatment of the SW480 colonic cell-line with *C. difficile* binary toxin (CDT, 1–100 ng/ml for 24 h) resulted in dose-dependent induction of **(A)** apoptosis and **(B)** inflammation, which was significantly attenuated with plantain NSP pre-treatment (at 10 mg/ml for 30 min). Apoptosis was assessed by caspase 3/7 Glo assay (RFU, relative fluorescence emission units), and pro-inflammatory cytokine IL-8 secreted to culture medium was assessed by ELISA. Data expressed as mean ± SD. Significant differences of toxin-treated cells from untreated controls; ^#^ *p* < 0.05, ^##^ *p* < 0.01, ^###^ *p* < 0.001 and between plantain NSP and vehicle pre-treated cells, *p < 0.05, **p < 0.01, ***p < 0.001; Kruskal-Wallis test; *N* = 2 experiments, *n* = 3 replicates.

Treatment of SW480 colonocytes with *C. difficile* binary toxin (CDT) dose-dependently increased cytotoxicity, as measured by enhanced caspase 3/7 activity, with peak response seen at 10 ng/ml CDT (283,890 ± 125,878 RFU) compared to untreated controls, 109,333 ± 35,941 (*p* < 0.05 Kruskal-Wallis test; *N* = 2, *n* = 3). Pre-treatment with plantain NSP inhibited the dose-dependent cytotoxicity induced by CDT, with peak reduction in toxin-treated SW480 colonocytes occurring at 100 ng/ml CDT (259,914 ± 67,103 RFU) which was reduced by >70% to 73,060 ± 53,767 (*N* = 2, *n* = 3; *p* < 0.001); see Figure 8A. Similarly, treatment with CDT also effected a dose-dependent increase in release of pro-inflammatory IL-8 from SW480 cells, with peak response seen at 100 ng/ml CDT (1041.92 ± 84.11 pg/ml/24 h) compared to untreated controls, 27.33 ± 10.81 pg/ml/24 h (*p* < 0.001). Again, it was seen that pre-treatment with plantain NSP inhibited the dose-dependent actions of CDT, with peak IL-8 release in toxin-treated SW480 colonocytes occurring at 100 ng/ml CDT reduced by approximately 50%, to 540.67 ± 41.21 pg/ml (*p* < 0.001); see Figure 8B.

CDT was also observed to induce release of inflammatory IL-8 from Intestine-407 cells, at test concentrations of 10 and 100 ng/ml over 24 h (both *p* < 0.001 one-way ANOVA; *N* = 2, *n* = 3). Pre-incubation of Intestine-407 cells with 10 mg/ml plantain NSP, reduced the amount of IL-8 released to the medium by both 10 and 100 ng/ml CDT (both *p* < 0.01; *N* = 2, *n* = 3 Kruskal-Wallis test). Plantain NSP was also effective in reducing Intestine-407 cytotoxicity induced by CDT (*p* < 0.05). No such inhibitory activity was seen with pre-treatment using 10 mg/ml oat NSP; see Supplementary Figure S6).

## 4 Discussion

Dietary fibre is widely recognised as being beneficial for intestinal health. A high dietary fibre intake increases faecal bulking and viscosity, resulting in shortened transit time through colon (Lattimer and Haub, 2010). Furthermore, its fermentation by resident gut microbiota produces short-chain fatty acids (SCFAs), such as butyrate, that act as an energy source for the intestinal epithelium (Kumar et al., 2020) and possess anti-carcinogenic properties (Chapkin et al., 2020). Fibre has also attracted considerable attention due to its role in the prebiotic effect, where it may selectively promote the growth of resident beneficial bacteria, through generation of butyrate to support intestinal health (Simpson and Campbell, 2015; Kumar et al., 2020). Here, we further suggest an alternative role of soluble dietary fibre as a “contrabiotic,” i.e. countering the action of microbes (Flanagan et al., 2011; Kumar et al., 2020).

In these studies, we provide additional evidence supporting the inhibitory effect of plantain NSP against diarrhoeal pathogen adhesion, specifically with respect to *C. difficile*. In further validation experiments, plantain NSP consistently inhibited the epithelial adherence of a range of clinically relevant *C. difficile* isolates. As these isolates varied according to their ribotype and toxin status, our results have suggested that the inhibitory activity of plantain NSP is likely independent of these two factors. We demonstrate that whilst a range of plant soluble fibres do significantly inhibit the epithelial adhesion of *C. difficile*, plantain NSP exhibits a particularly strong inhibitory effect. This is consistent with its superior blockade of adherence and invasion by a range of other pathogens including various *E. coli* and *Salmonella* species. Other evidence supporting a protective effect of plantain against diarrhoeal disease includes randomised controlled trials in which the juice from boiled green bananas has been shown to be effective in a variety of diarrhoeal diseases (Rabbani et al., 2001; Rabbani et al., 2004; Alvarez-Acosta et al., 2009).

Here, we also provide further insight into the mechanism of the inhibitory action of plantain soluble non-starch polysaccharides. We present data indicating that plantain soluble dietary fibre likely exerts its inhibitory effect against *C. difficile via* an interaction with the intestinal epithelium, rather than through interaction with bacterial adhesins. This is similar to the mechanism we have previously reported for plantain NSP blockade of *Salmonella* spp. adhesion with, and invasion into, intestinal epithelial cells (Parsons et al., 2014). Ussing chamber experiments had also shown that pre-treatment of human terminal ileum with plantain NSP generated an increase in transmucosal short circuit current (I_sc_), indicative of electrogenic chloride ion efflux and associated water transport (Parsons et al., 2014). Epithelial secretion of fluid is already recognised as a form of innate immunity, thought to serve as a non-specific method of clearing bacteria and their toxins from the intestinal mucosa (Keely et al., 2011). Therefore, it is plausible that plantain NSP could act in this manner to reduce *C. difficile* epithelial adhesion. We found that plantain NSP increased chloride ion efflux from Caco-2 cell monolayers in a dose-dependent manner. Due to limited available quantities of other dietary plant NSP sources, we could only test for their ability to induce epithelial cell chloride ion channel activity at a dose of 5 mg/ml. Compared to plantain NSP at this level, which showed approximately 70% inhibition of bacterial adhesion, and 4-fold enhancement of chloride ion channel activity, other dietary plant NSP sources, shown not to exhibit an inhibitory effect on bacterial adhesion, had weaker, or no such, activity. However, whilst we showed that elevated epithelial cell chloride ion channel activity induced by plantain NSP could be significantly suppressed in the presence of chloride ion channel antagonists, under these same conditions, plantain NSP still possessed significant ability to reduce *C. difficile*-epithelial cell adhesion. Further insight into the action of plantain NSP on key chloride ion transporters in intestine and colon, coupled with *in vitro* bacterial adhesion assays will be necessary.

In our *in vitro* studies presented here, only soluble NSP from one dicot—broccoli—and from the non-commelinoid monocot leek showed inhibitory effects against *C. difficile*-epithelial interactions comparable to plantain NSP (a commelinoid monocot). NSP from the other eight dicots tested and from the commelinoid monocot oat were mainly ineffective, or showed low efficacy at best. That the desired inhibitory effects can be obtained by utilising NSP from dicots, commelinoid and non-commelinoid monocots suggests that efficacy is not related to cell wall type. Dicots and non-commelinoid monocots have type I cell walls, formed from a cross-linked but relatively open network of cellulose and xyloglucans, with smaller amounts of linked arabinoxylans, glucomannans and galactoglucomannans (Vogel, 2008; Pattathil et al., 2015). A pectin-based matrix envelops the cellulose-xyloglucan network, contributing up to one third of the cell wall dry weight. Pectins can be sub-divided to three distinct groups; homogalacturonan and rhamnogalacturonans, RGI and RGII (Fry, 2004; Atmodjo et al., 2013). These polymers are covalently linked, with the rhamnogalacturonans typically containing a high proportion of neutral sugars, such as arabinose, rhamnose and galactose (Fry, 2011). Commelinoid monocots have type II cell walls; these are composed of a tightly packed network of glucuronoarabinoxylans, mixed-link glucans such as β-glucans, and a relatively larger amount of cellulose, compared to type I walls (Cui and Wang, 2009). Type II cell walls are relatively pectin-poor, especially in the Poaceae, but pectins are known to occur in higher amounts in bananas and plantain. Thus, it is evident that the inhibitory NSP preparations discovered in our work, deriving from different cell wall types, could have very different polysaccharide compositions. From the data discussed, it is difficult to discover whether the inhibition of *C. difficile*-epithelial interactions by plantain, broccoli and leek NSP has a common cause, or whether the observed effects are due to completely different polysaccharide interactions.

However, in further work presented here, the inhibitory activity of plantain NSP against *C. difficile-*epithelial interaction is shown to be due to the acidic (or pectin-rich) fraction within this particular soluble dietary fibre, whilst little or no inhibition occurred with the neutral fraction (the larger proportion of total NSP). These results also support observed blockade of *Salmonella* Typhimurium to intestinal epithelial cells with the acidic fraction of plantain NSP (Parsons et al., 2014). The pectin component of plantain NSP is known to contain significant quantities of homogalacturonan, and much smaller amounts of RG-I; our previous studies have shown that the inhibitory activity associated with the acidic fraction of plantain NSP is linked to the homogalacturonan component (no RG-I/RG-II was detected in the active fraction) (Parsons et al., 2014). This is interesting, because water-soluble pectin from broccoli is reported to be composed of unbranched, highly-esterified pectin (homogalacturonan) alongside less abundant pectic polymers with side-chains that are less esterified (Christiaens et al., 2011), and leek pectin is reported to consist largely of relatively high methyl-esterified homogalacturonan with smaller quantities of RG-I decorated with galactose-rich side chains (Ognyanov et al., 2020). In contrast, pectins from the dicots which did not show significant inhibitory activity against *C. difficile*-epithelial interactions - strawberry, blueberry, tomato, celery, runner bean, pear and apple—have been reported to contain both RG-I and II subtypes in significant proportions, with less unbranched homogalacturonan (Lefever et al., 2004; Wu et al., 2020; Hotchkiss et al., 2021); and oat NSP contains hardly any pectins at all. It is possible that the homogalacturonan content of plantain, broccoli and leek NSP may be a commonality which underlies the inhibitory activity shown, although in the current absence of data on broccoli and leek NSP this is clearly a speculative hypothesis. In future work, it will be interesting to examine whether the proportion of homogalacturonan in pectic polysaccharides has a direct bearing on NSP inhibitory potential. There is currently a strong focus on RG-I as a source of dietary polysaccharides beneficial to health (Wu et al., 2020), but pectic polysaccharides are among the most complex in the plant cell wall and our *in vitro* data shows that other possibilities should not be overlooked.

Other dietary factors rich in complex oligosaccharides, such as human milk, are also well established as being able to prevent bacteria-host epithelium interactions (Asadpoor et al., 2020), inhibiting adhesion of bacteria such as *Campylobacter jejuni* and *Salmonella enterica* (Asadpoor et al., 2020), diffusely adherent *E. coli* (DAEC) (Nascimento de Araújo and Giugliano, 2000) and enteroaggregative *E. coli* (EAEC) (Nascimento de Araújo and Giugliano, 2000). More specifically, pectic oligosaccharides have also been demonstrated to inhibit *C. jejuni* intestinal epithelial adherence *in vitro* (Ganan et al., 2010). In addition, pectin from ginseng has been shown to possess anti-adhesive activity against other gut pathogens such as *Helicobacter pylori*, and some ability to inhibit haemagglutination by bacteria, including that caused by *Staphylococcus aureus* and *Propionibacterium acnes* (Lee et al., 2006a). An acidic polysaccharide extract from green tea (*Camellia sinensis*) has also been shown to possess inhibitory activity, blocking adhesion of *H. pylori,* as well as *S. aureus* and *P. acnes* (Lee et al., 2006b).

It is a reasonable assumption that ingested soluble fibre from plantain should pass through the human small intestine intact. Given a typical passage of approximately 1 L of fluid per day into the caecum, a daily intake of 10 g of soluble fibre should therefore result in a concentration of 10 mg/ml, as tested here, in the caecum but it is then likely to be fermented quite rapidly (Roberts et al., 2010). In keeping with this it is notable that the large EPIC cohort study showed a protective effect for dietary fibre against proximal but not distal colon cancer (Murphy et al., 2012) and the Nurses’ Health cohort study has shown a protective effect for fruit fibre against Crohn’s disease, which typically affects the ileum and caecum, but not against ulcerative colitis, which typically affects the distal colon and rectum (Ananthakrishnan et al., 2013). It is plausible though that the rate of fermentation of plantain fibre might be considerably diminished during diarrhoeal disease thus allowing a protective effect in the distal colon.

Although controversy exists as to whether *C. difficile* epidemic strains do actually have significantly high sporulation rates (Akerlund et al., 2008; Burns et al., 2010; Merrigan et al., 2010), studies suggest that *C. difficile* spores can in fact persist within the intestinal tract due to their ability to interact with, and adhere to, the intestinal epithelium (Paredes-Sabja and Sarker, 2012). In our studies, we observed that 85% of *C. difficile* spores adhered to human intestinal Caco-2 cells, in comparison to only 4% of *C. difficile* vegetative cells. Our results are in agreement with those of previous studies, which have also shown that *C. difficile* spores are much more adhesive to monolayers of Caco-2 cells (Dingle et al., 2010; Paredes-Sabja and Sarker, 2012) and provide further evidence to suggest that spore adherence might be exploited by *C. difficile* as a means of persistence in the host. It is worth noting, that the purification procedure to obtain *C. difficile* spores includes treatment with proteinase K, removing proteins from the external exosporium layer (Escobar-Cortés et al., 2013). This not only enhances adherence of *C. difficile* spores, both to inert surfaces and to intestinal epithelial cells in culture, including Caco-2, but also increases colony forming efficiency (Escobar-Cortés et al., 2013). It is speculated that the exosporium acts physically to slow escape of a new vegetative cell from a germinated spore so as to allow for correct timing of outgrowth and colonisation of the intestinal epithelium (Escobar-Cortés et al., 2013). The low level of interaction of vegetative cells with intestinal cell monolayers in culture, in comparison to that seen for spores, might also reflect that *C. difficile*, although able to germinate in an aerobic environment, cannot grow and divide in the presence of the levels of oxygen seen for cell-line monolayer culture (Sorg and Sonenshein, 2010), albeit in our infection assays, exposure was relatively short-term, over 2 h. Recent bioengineered 3D human intestinal tissue models that recapitulate the lower O_2_ conditions produced in the lumen of these tissues (Shaban et al., 2018) may better support longer term studies of *C. difficile* vegetative cell and spore-epithelium interactions under anaerobic conditions. Perhaps more importantly, our results demonstrate that soluble NSP from plantain significantly inhibits the epithelial adhesion of *C. difficile* spores. Persistence of *C. difficile* spores within the colon of CDI patients not only complicates treatment options, but also increases recurrence rates associated with the disease (Castro-Córdova et al., 2021) and increases potential to transmit *C. difficile* to other patients, even after diarrhoea has resolved (Crobach et al., 2018). Indeed, 25–85% of all CDI recurrences are attributed to the *C. difficile* strain that caused initial infection (Oka et al., 2012). Our results are therefore very promising with respect to the therapeutic potential of plantain soluble dietary fibre and suggest that its use as a dietary supplement could also help maintain remission from recurrent CDI.

As *C. difficile* and its toxins both interact with the intestinal epithelium and possess lectin activity, we hypothesised that it was highly likely that soluble NSP from plantain could also inhibit the action of *C. difficile* TcdA, TcdB and CDT on the intestinal epithelium. Previous studies monitoring the mono-glucosylation of Rho-GTPases have focussed on Rac1 due to the fact that its inactivation has been deemed the main mechanism responsible for the cytopathic effects of the *C. difficile* toxins TcdA and TcdB (Popoff and Geny, 2011). TcdA and TcdB mediated Rac1 mono-glucosylation has previously been investigated in HT29 colonocytes (Gerhard et al., 2008) through monitoring the level of cellular Rac1 using anti-Rac1 102 antibody, which has impaired recognition of Rac1 following its mono-glucosylation, at Threonine position 35, giving an apparent decrease in Rac1 levels. In our studies, we found that treatment of Caco-2 cells with 10 ng/ml *C difficile* TcdA or TcdB resulted in increased cellular Rac1 mono-glucosylation, as measured by a decrease in anti-Rac1 102 antibody binding. We observed that intracellular TcdA and TcdB mediated activity were both unaffected by pre-treatment with soluble NSP from plantain. Other studies have provided evidence of small molecules from natural sources with activity against *C. difficile* (Tam et al., 2015; Pal and Seleem, 2020), including some based on tea derivatives and flavonoids that can directly reduce glucosylating *C. difficile* toxin-induced cellular damage likely through blockade of glucosyltransferase and hydrolase activity (Tam et al., 2015), highlighting the important need to perhaps consider combination non-antibiotic based therapies for treatment, and prophylactic prevention, of CDI.

Other *C. difficile* toxin-mediated effects include the induction of the pro-inflammatory response (Kim et al., 2006; Zemljic et al., 2010), as well as the activation of apoptosis (Gerhard et al., 2008). In our studies, treatment of Caco-2 cells with TcdA or TcdB increased cellular IL-8 release (or KC, from mouse organoids) and caspase-3 activation in a dose-dependent manner. Interestingly, the cellular toxin response was slightly higher in Caco-2 cells that were treated with TcdA. Indeed, there has been great debate about the individual importance of TcdA and TcdB during the course of clinical infection, and many conflicting studies exist. Whilst our results are in agreement with studies that suggest TcdA exhibits higher potency and cytotoxicity *in vitro* (Poutanen and Simor, 2004; Kuehne et al., 2010), others highlight that TcdB is the more potent toxin (Shen, 2012). *In vivo* animal experiments have also created debate over the importance of TcdA and TcdB; whilst TcdA^+^, TcdB^−^ isogenic mutants used in a study by Keuhne and colleagues were able to induce an *in vivo* pathogenic effect (Kuehne et al., 2010), in a study by Lyras *et al.*, the equivalent mutant strains were observed to be avirulent (Lyras et al., 2009). TcdA^−^, TcdB^+^ *C. difficile* strains are routinely isolated from CDI patients and are capable of causing extensive disease (Voth and Ballard, 2005), whilst very few TcdA^+^, TcdB^−^ strains have been reported (Kuehne et al., 2010). This suggests that both TcdA and TcdB likely play an important role in the pathogenesis of CDI. Our results demonstrate that whilst infection of Caco-2 cells with a TcdA^−^, TcdB^+^ *C. difficile* strain does mount a considerable cellular response, the response is much higher when cells are treated with a TcdA^+^, TcdB^+^ *C. difficile* strain, suggesting that they could in fact work together in synergy to produce an additive effect. For CDT, we showed in our studies, that this binary toxin was very potent in inducing pro-inflammatory IL-8 release from human SW480 colonocytes and Intestine-407 cells.

Interestingly, our results indicate that plantain NSP reduces the evoked cellular IL-8 response and caspase-3 activation in cells treated either with purified TcdA, TcdB or CDT, as well as those infected with toxin positive and toxin negative *C. difficile* strains. Basal responses were also observed to be reduced in cell-lines pre-treated with plantain NSP, suggesting that soluble dietary fibre, and particularly its acidic components, may have a general protective effect against the induction of a pro-inflammatory response, cellular damage and apoptosis. As plantain NSP seems to have no direct effect on toxin-mediated mono-glucosylation of Rac1, rather it has a more general effect against the cellular induction of the pro-inflammatory response and apoptosis, these studies collectively indicate that plantain NSP does not inhibit the specific intracellular activity of *C. difficile* toxins at intestinal epithelial cells. Close proximity of *C. difficile* to the host epithelium is almost certainly necessary for toxin release (Sears and Kaper, 1996) and preventing these interactions should therefore be of therapeutic benefit. With plantain NSP preventing adhesion, and close apposition of, *C. difficile* to the intestinal epithelium, we speculate that toxins would likely not be released to any great extent, to be internalised by host cells.

## 5 Conclusion

In summary, plantain soluble non-starch polysaccharides possess the ability to disrupt the epithelial interactions of *C. difficile* vegetative cells and spores. This suggests that dietary supplementation with plantain soluble fibre may confer a protective effect in CDI patients by preventing bacterial adhesion to the mucosa, i.e. a “contrabiotic” effect. More specifically, plantain NSP blocks *C. difficile* adhesion, with inhibitory activity against *C. difficile* found within the acidic (pectin-rich) polysaccharide component, through interaction with the intestinal epithelium. Further studies are required to confirm the inhibitory epithelial mediated actions of plantain NSP in further *in vitro* models, and relevant *in vivo* models (Best et al., 2012; Hutton et al., 2014; Pruss and Sonnenburg, 2021).

## Data Availability

The original contributions presented in the study are included in the article/Supplementary Material, further inquiries can be directed to the corresponding author.

## Supplementary Material

Supplementary Figure S1 Visualisation of purified *C.* *difficile spores.*

Supplementary Figure S2 Soluble plantain NSP acts on the epithelium to inhibit the interaction of diarrhoeal pathogen *C. difficile.*

Supplementary Figure S3 Plantain NSP induces cellular chloride ion channel activity in intestinal cells in a dose-dependent manner, but inhibition of chloride efflux does not inhibit plantain NSP-induced blockade of *C. difficile*-epithelial cell adhesion.

Supplementary Figure S4 Treatment of murine intestinal organoids with *C. difficile* toxins A (TcdA) and B (TcdB) induces cell rounding.

Supplementary Figure S5
*C. difficile* toxins A (TcdA) and B (TcdB) mediate mono-glucosylation of intracellular Rac1 in intestinal epithelial cells.

Supplementary Figure S6 Soluble plantain NSP reduces C. *difficile* binary toxin mediated inflammation, and cytotoxicity in Intestine-407 cells.

Supplementary Table S1 Compositional data of soluble non-starch polysaccharides (NSP) derived from dietary sources.

Supplementary Table S2 Sporulation characteristics of C. difficile clinical isolates.
