# Supplementary material for: Soluble Non-Starch Polysaccharides From Plantain (Musa x paradisiaca L.) Diminish Epithelial Impact of Clostridioides difficile
Source: Front Pharmacol. 2021 Dec 10;12:766293. doi: 10.3389/fphar.2021.766293 (PMC8707065; doi:10.3389/fphar.2021.766293)

Supplementary materials:

Figure S5. ***Clostridioides difficile*** **toxins A (TcdA) and B (TcdB) mediate mono-glucosylation of intracellular Rac1 in intestinal epithelial cells.** Immunoblot analysis illustrates an increase in mono-glucosylation of the small GTP-binding protein Ras-related C3 botulinum toxin substrate (Rac1) in Caco2 cells following treatment with 10 ng/mL *C. difficile* toxins, **(A)** TcdA for 24 h, and **(B)** TcdB for 48 h. Protein bands were analysed by densitometry. Mono-glucosylated Rac1 levels (assessed by antibody clone 102) were normalised to total Rac1 (assessed by antibody 238a). Representative blots shown from N=3 experiments, n=3 replicates. Significant differences from untreated control, * $P < 0.05$, ** $P < 0.01$; Kruskal Wallis test).

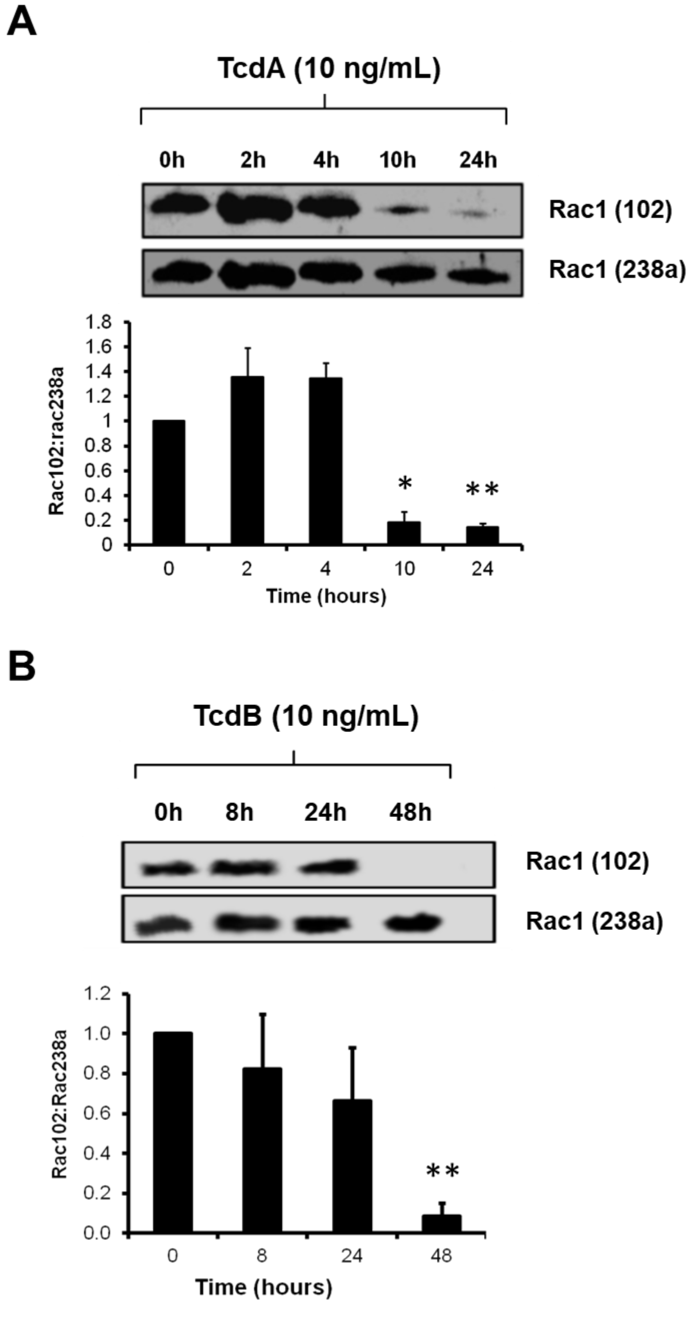

Supplement: Supplementary file 1 [file Image5.pdf]
