# Supplementary material for: Soluble Non-Starch Polysaccharides From Plantain (Musa x paradisiaca L.) Diminish Epithelial Impact of Clostridioides difficile
Source: Front Pharmacol. 2021 Dec 10;12:766293. doi: 10.3389/fphar.2021.766293 (PMC8707065; doi:10.3389/fphar.2021.766293)
Supplement: Supplementary file 2 [file Table1.DOCX]

**Supplementary materials:**

**Table S1: Compositional data of soluble non-starch polysaccharides (NSP) derived from other dietary sources.**

| **NSP source*** | **Soluble NSP % cell wall dry matter** | **Uronic acids**  **(μg/mg NSP)** | **Total quantified monosaccharides**  **(μg/mg NSP)** |  | **Monosaccharide composition^#^**  **(μg/mg NSP)** | | | | | | |  | **Monosaccharide composition**  **(% total quantified monosaccharides)** | | | | | | |
| --- | --- | --- | --- | --- | --- | --- | --- | --- | --- | --- | --- | --- | --- | --- | --- | --- | --- | --- | --- |
|  |  |  |  |  | **Rha** | **Fuc** | **Rib** | **Ara** | **Man** | **Gal** | **Glu** |  | **Rha** | **Fuc** | **Rib** | **Ara** | **Man** | **Gal** | **Glu** |
| **Apple** | 52.9 | 199.0 | 184.2 |  | 31.8 | 0.0 | 0.0 | 60.7 | 22.8 | 32.2 | 36.8 |  | 17.2 | 0.0 | 0.0 | 33.0 | 12.3 | 17.5 | 20.0 |
| **Banana** | 35.0 | 438.9 | 132.2 |  | 14.3 | 0.0 | 0.0 | 42.5 | 8.6 | 44.4 | 22.4 |  | 10.8 | 0.0 | 0.0 | 32.2 | 6.5 | 33.6 | 16.9 |
| **Bean (runner)** | 41.8 | 316.5 | 269.4 |  | 27.7 | 0.0 | 0.0 | 175.2 | 18.0 | 27.8 | 20.7 |  | 10.3 | 0.0 | 0.0 | 65.0 | 6.7 | 10.3 | 7.7 |
| **Blueberry** | 37.9 | 192.1 | 387.3 |  | 62.6 | 0.0 | 0.0 | 140.5 | 47.0 | 55.3 | 81.9 |  | 16.2 | 0.0 | 0.0 | 36.3 | 12.1 | 14.3 | 21.1 |
| **Broccoli** | 29.4 | 174.8 | 192.5 |  | 11.9 | 0.0 | 0.0 | 26.4 | 22.7 | 78.4 | 53.1 |  | 6.2 | 0.0 | 0.0 | 13.7 | 11.8 | 40.7 | 27.6 |
| **Celery** | 20.8 | 457.6 | 210.6 |  | 24.2 | 0.0 | 0.0 | 96.6 | 15.5 | 66.2 | 8.0 |  | 11.5 | 0.0 | 0.0 | 45.9 | 7.4 | 31.4 | 3.8 |
| **Leek** | 31.1 | 230.1 | 577.8 |  | 113.7 | 0.0 | 0.0 | 41.0 | 81.2 | 13.9 | 328.0 |  | 19.7 | 0.0 | 0.0 | 7.1 | 14.1 | 2.4 | 56.8 |
| **Oat** | 36.9 | 24.4 | 436.7 |  | 0.4 | 0.2 | 0.0 | 10.7 | 3.1 | 10.8 | 387.1 |  | 0.1 | 0.0 | 0.0 | 2.5 | 0.7 | 2.5 | 88.6 |
| **Pear** | 42.7 | 327.4 | 91.6 |  | 14.3 | 0.0 | 0.0 | 14.3 | 19.1 | 13.5 | 30.5 |  | 15.6 | 0.0 | 0.0 | 15.6 | 20.8 | 14.7 | 33.2 |
| **Pepper**  **(red bell)** | 42.4 | 358.5 | 367.8 |  | 45.4 | 0.0 | 0.0 | 66.5 | 110.5 | 28.2 | 117.1 |  | 12.3 | 0.0 | 0.0 | 18.1 | 30.1 | 7.7 | 31.9 |
| **Plantain (green)** | 42.3 | 169.1 | 370.9 |  | 70.8 | 0.0 | 0.0 | 59.6 | 84.2 | 14.8 | 141.6 |  | 19.1 | 0.0 | 0.0 | 16.1 | 22.7 | 4.0 | 38.2 |
| **Strawberry** | 40.7 | 304.2 | 232.7 |  | 59.3 | 0.0 | 0.0 | 124.3 | 11.7 | 21.1 | 16.4 |  | 25.5 | 0.0 | 0.0 | 53.4 | 5.0 | 9.1 | 7.0 |
| **Tomato** | 50.2 | 299.4 | 591.3 |  | 70.1 | 0.0 | 0.0 | 296.9 | 43.8 | 84.6 | 95.8 |  | 11.9 | 0.0 | 0.0 | 50.2 | 7.4 | 14.3 | 16.2 |

*water soluble, 80% ethanol insoluble fibre.

#Monosaccharide residues: Rha, rhamnose; Fuc, fucose; Rib, ribose; Ara, arabinose; Man, mannose; Gal, galactose; Glu, glucose
