# Supplementary material for: Soluble Non-Starch Polysaccharides From Plantain (Musa x paradisiaca L.) Diminish Epithelial Impact of Clostridioides difficile
Source: Front Pharmacol. 2021 Dec 10;12:766293. doi: 10.3389/fphar.2021.766293 (PMC8707065; doi:10.3389/fphar.2021.766293)

Supplementary materials:

Figure S6. Soluble plantain NSP reduces *Clostridioides difficile* binary toxin mediated inflammation, and cytotoxicity in Intestine-407 cells. Cell monolayers were treated with *C. difficile* binary toxin (CDT), at 10 and 100 ng/mL for 24h, in the absence (black bars) or presence of 10 mg/mL plantain NSP (white bars) or oat NSP (grey bars). Media harvested from cells was analysed for release of **(A)** IL-8 (by ELISA) and **(B)** cytotoxicity marker adenylate kinase (by Toxilight bioassay). Significant increases in inflammation and cytotoxicity in CDT-treated cells versus untreated controls; ##$P < 0.01$, ###$P < 0.001$, ####$P < 0.0001$; one-way ANOVA, with Dunnett's post-hoc test. Plantain NSP significantly reduced CDT-mediated actions on Intestine-407 cells; * $P < 0.05$, ** $P < 0.01$, *** $P < 0.001$). No similar blockade was seen with oat NSP. N=2 experiments, n=3 replicates. No significant changes were seen in protein levels in cell lysates measured by bicinchoninic acid (BCA) assay.

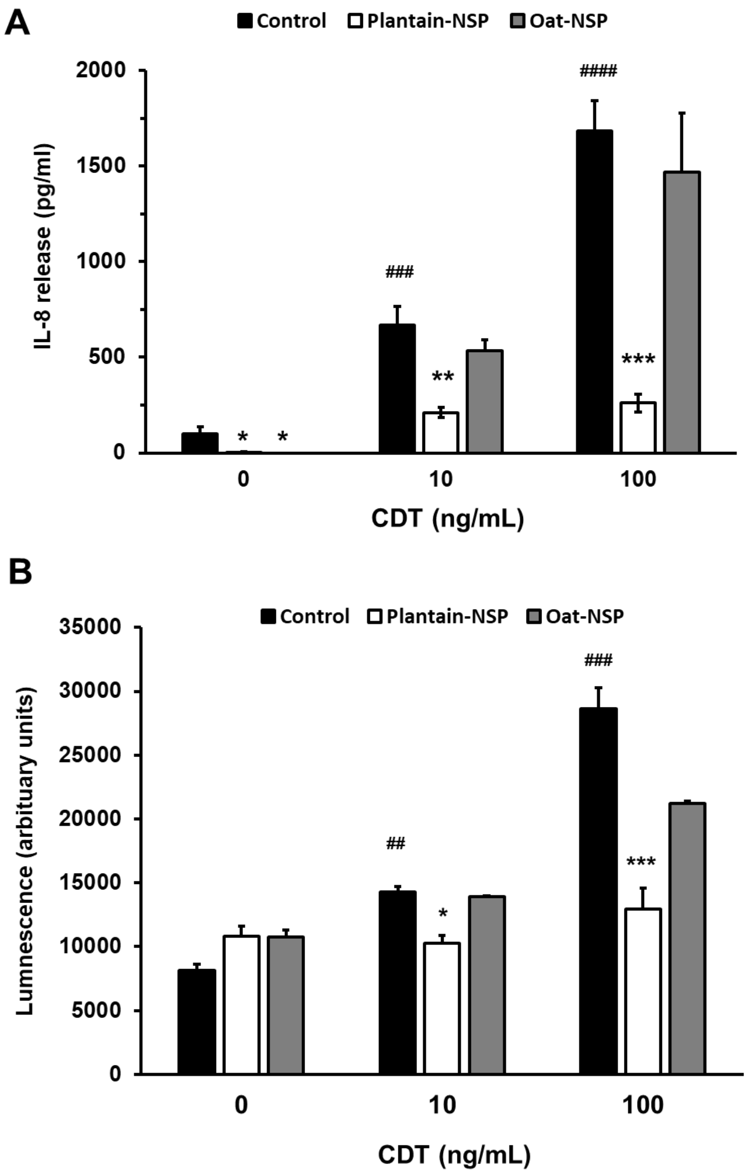

Supplement: Supplementary file 3 [file Image6.pdf]
