# Supplementary material for: Soluble Non-Starch Polysaccharides From Plantain (Musa x paradisiaca L.) Diminish Epithelial Impact of Clostridioides difficile
Source: Front Pharmacol. 2021 Dec 10;12:766293. doi: 10.3389/fphar.2021.766293 (PMC8707065; doi:10.3389/fphar.2021.766293)

Supplementary materials:

Figure S4. Treatment of murine intestinal organoids with *Clostridioides difficile* toxins A (TcdA) and B (TcdB) induce epithelial cytotoxicity. (A) Growth in culture of stem cell derived 3-D intestinal organoids from C57BL/6J mice. Representative brightfield images showing a time course of the growth from an isolated crypt grown in Matrigel and cultured for 6 days. White arrow heads indicate Paneth cells. Scale bar = 100µm. Organoids were passaged at day 7 and re-grown for 4d, then treated for 24 h with *C. difficile* TcdA and TcdB. **(B)** Images showing morphological changes induced by 100 ng/mL TcdA and TcdB similar to that seen following treatment with 100 ng/mL tumour necrosis factor alpha (TNFα), a positive control for induction of organoid rounding, indicative of epithelial cytotoxicity. **(C)** Dose-dependent induction of cytotoxicity following treatment with 1 to 100 ng/mL TcdA and TcdB was quantified, with organoids assessed for increased circularity (a value of 1.0 equating to a full circle) determined using Image J software (N=4). Significant differences compared to untreated controls, $^{**}P < 0.01$, $^{***}P < 0.001$; one-way ANOVA, with Dunnett's post-hoc comparisons of treatment means.

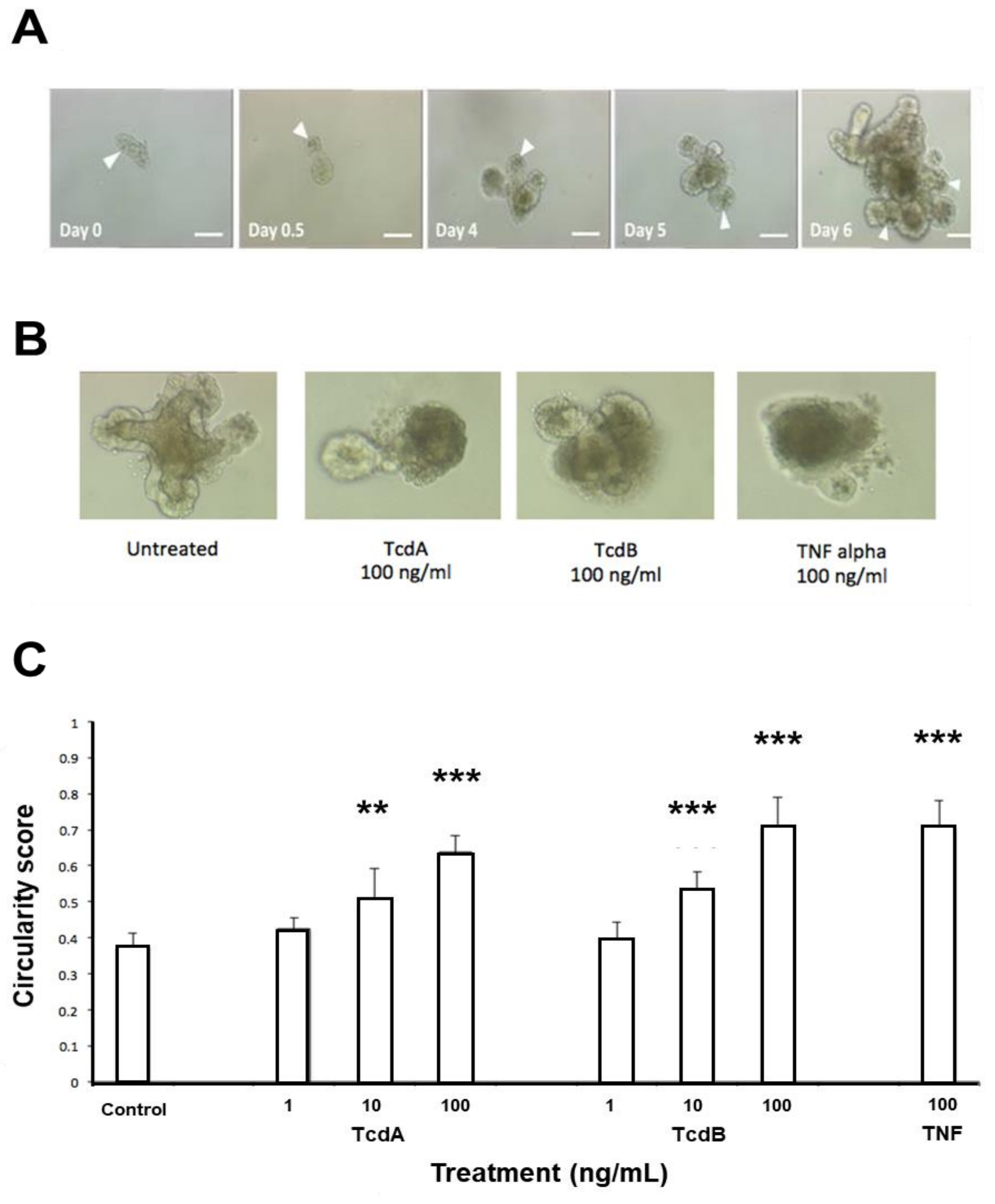

Supplement: Supplementary file 4 [file Image4.pdf]
