# Supplementary material for: Soluble Non-Starch Polysaccharides From Plantain (Musa x paradisiaca L.) Diminish Epithelial Impact of Clostridioides difficile
Source: Front Pharmacol. 2021 Dec 10;12:766293. doi: 10.3389/fphar.2021.766293 (PMC8707065; doi:10.3389/fphar.2021.766293)

Supplementary materials:

Figure S2. Soluble plantain NSP acts on the epithelium to inhibit the interaction of diarrhoeal pathogen *Clostridioides difficile*. Plantain NSP blockade of adhesion of *C. difficile* 80042 (a toxin A/toxin B/binary toxin positive clinical isolate) to Caco2 cells was assessed under different pre-treatment conditions: **(A)** Standard pre-treatment of cell monolayers with plantain NSP (30 min), followed by infection for 2 h. **(B)** Pre-treatment of cell monolayers with soluble plantain NSP (30 min), followed by removal from monolayers with three washes of sterile PBS pH 7.3 prior to infection for 2 h. **(C)** Pre-treatment of a suspension of *C. difficile* with plantain NSP (30 min), followed by centrifugation, resuspension of bacteria in antibiotic-free media and infection for 2 h. Adhesion is expressed relative to CFU/mL found in untreated control (set as 100%). N≥1 experiments, n=3 replicates. Significant differences from untreated control ** $P < 0.01$, *** $P < 0.001$; Kruskal Wallis test).

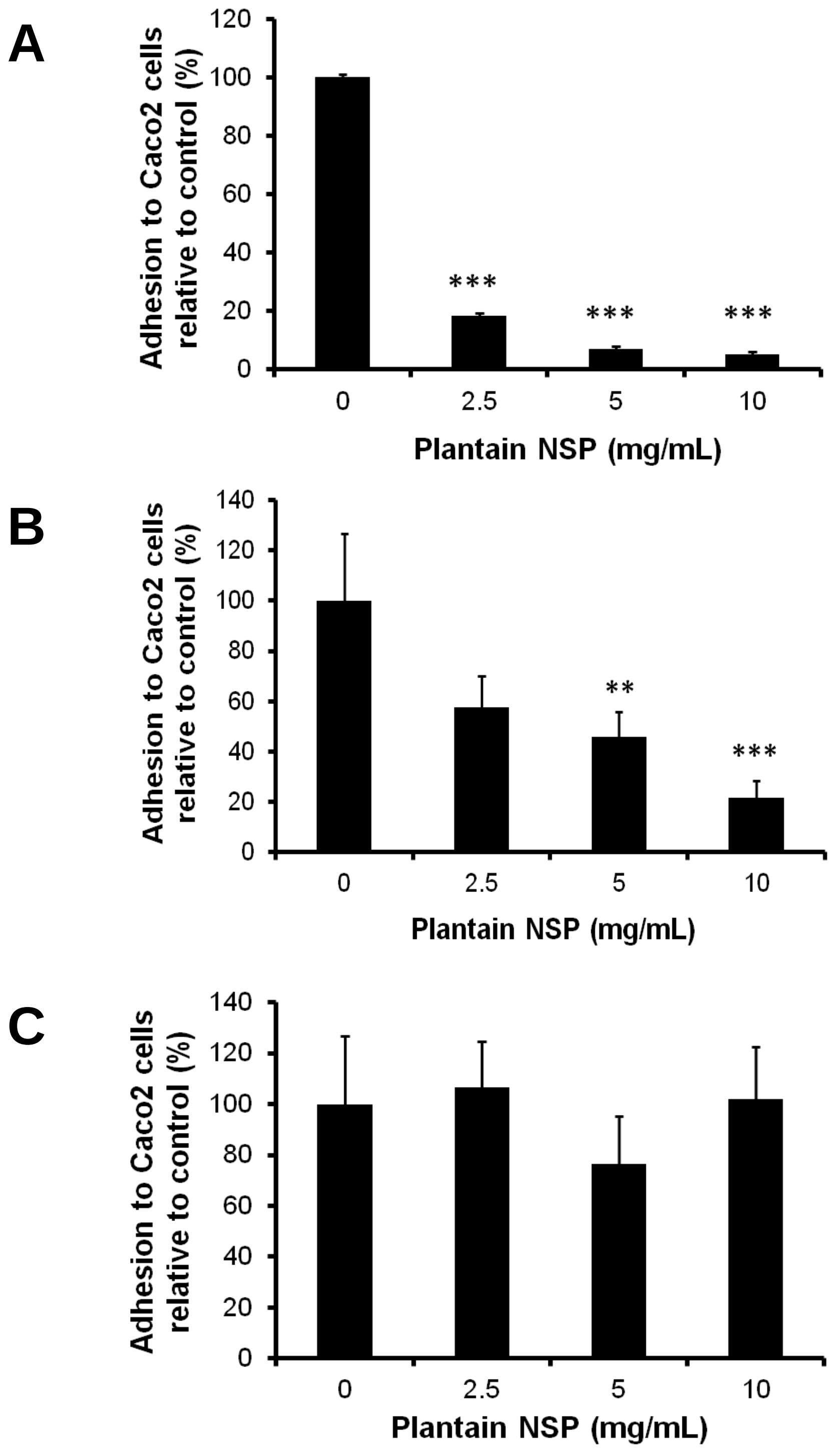

Supplement: Supplementary file 5 [file Image2.pdf]
