# Supplementary material for: Soluble Non-Starch Polysaccharides From Plantain (Musa x paradisiaca L.) Diminish Epithelial Impact of Clostridioides difficile
Source: Front Pharmacol. 2021 Dec 10;12:766293. doi: 10.3389/fphar.2021.766293 (PMC8707065; doi:10.3389/fphar.2021.766293)
Supplement: Supplementary file 6 [file Image3.pdf]

Supplementary materials:

Figure S3. Plantain NSP induces cellular chloride ion channel activity in intestinal cells in a dose-dependent manner, but inhibition of chloride efflux does not inhibit plantain NSP-induced blockade of *Clostridioides difficile*-epithelial cell adhesion. (A) Pre-treatment for 30 min with soluble plantain NSP (2.5-20 mg/mL) induced chloride channel activity in Caco2 cells. Oat and bean NSP, at 5 mg/mL, also significantly increased chloride channel activity, whilst pear, pepper and apple NSP exhibited no significant effect. Chloride channel activity in was measured by monitoring cellular iodide efflux measured using the Modified Sandell-Kolthoff reaction. Chloride channel activity was expressed as fold change in comparison to the vehicle treated control. **(B)** Soluble plantain fibre (10 mg/mL) mediated cellular chloride channel activity was dose-dependently reduced in the presence of chloride channel antagonists, NPPB (0 – 800 μM) and CFTR Inh172 (0 – 200 μM) compared to vehicle-treated controls**. (C)** The action of plantain NSP on *C. difficile* epithelial cell adhesion was not seen to be blocked with 800 μM NPPB or 200 μM Inh172 (1 h pre-treatment, prior to treatment with or without plantain NSP (30 min)). **(D)** *C. difficile* adhesion assessed following pre-treatment of Caco2 cells with or without 800 μM NPPB or 200 μM Inh172 (1 h), followed by treatment with or without 100 μM RP107 (30 min). For all, bacterial adhesion is expressed relative to the CFU/mL found in the untreated control (set as 100%), with N=3 experiments, n ≥ 3 replicates; ** $P < 0.01$, *** $P < 0.001$; Kruskal-Wallis test.

A

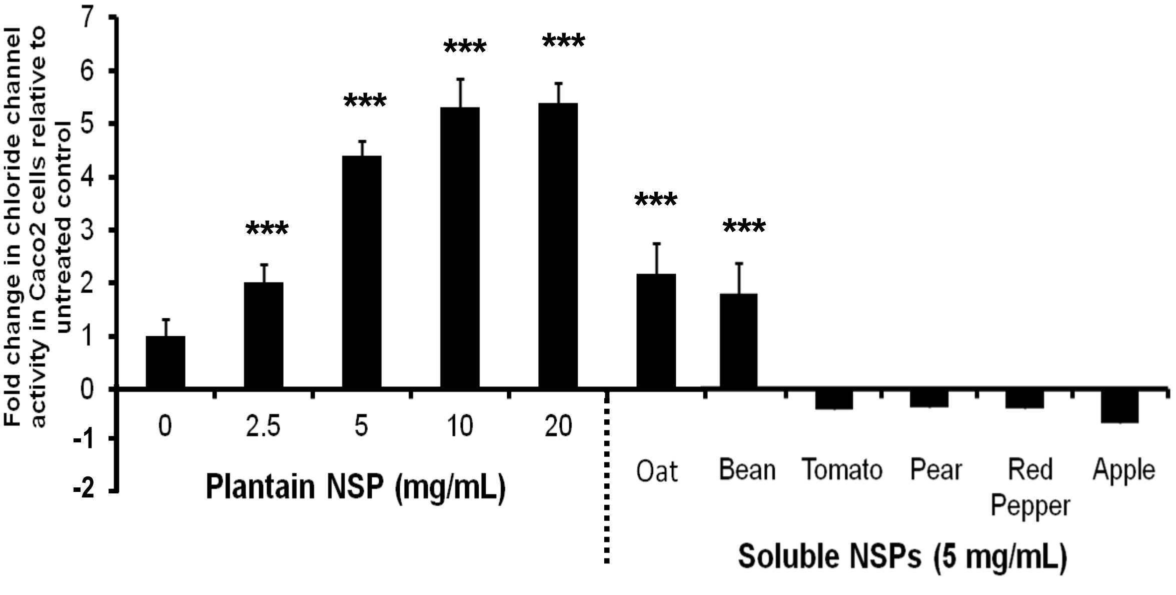

B

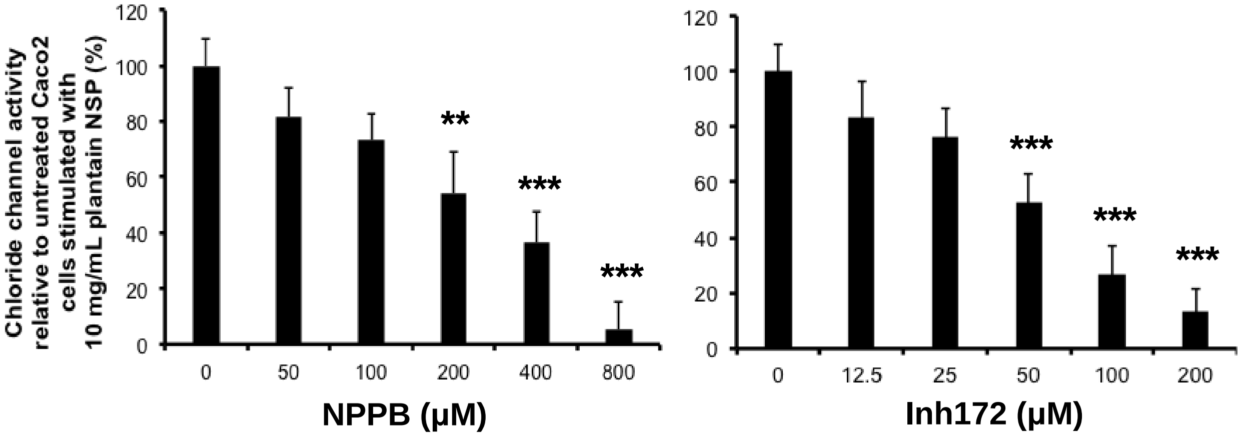

C

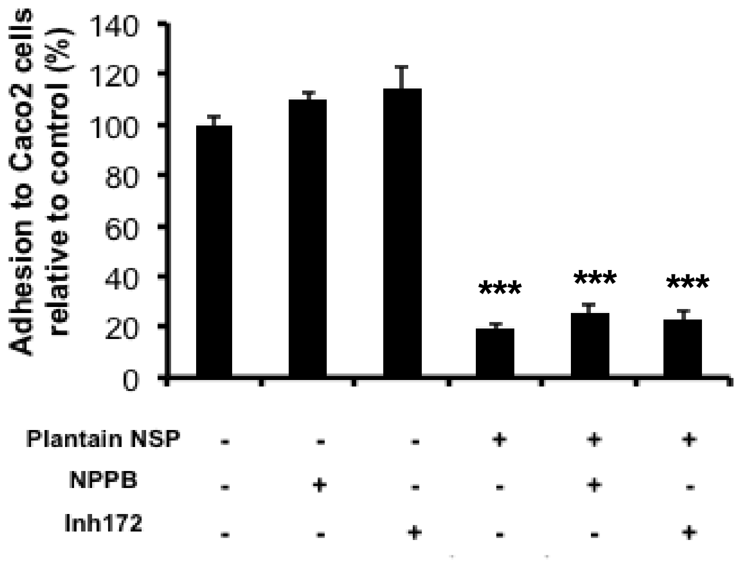
Adhesion to Caco2 cells relative to control (%)
140
120
100
80
60
40
20
0
***
***
***
Plantain NSP - - - + + +
NPPB - + - - + -
Inh172 - - + - - +

D

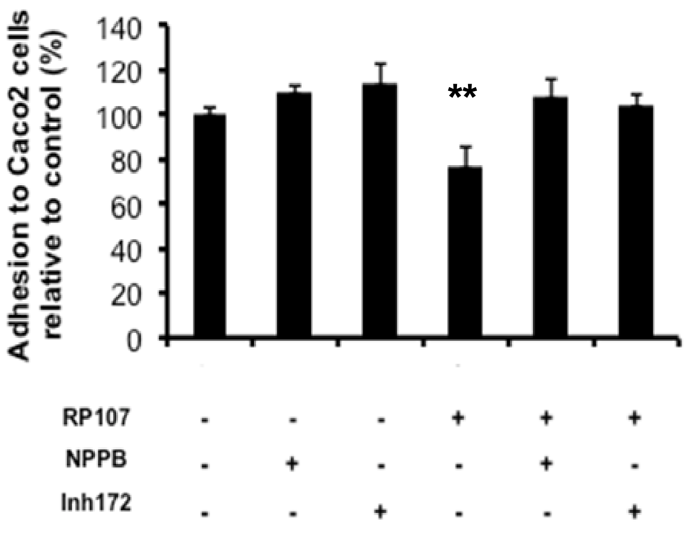
Adhesion to Caco2 cells relative to control (%)
140
120
100
80
60
40
20
0
**
RP107 - - - + + +
NPPB - + - - + -
Inh172 - - + - - +
