# Supplementary material for: Soluble Non-Starch Polysaccharides From Plantain (Musa x paradisiaca L.) Diminish Epithelial Impact of Clostridioides difficile
Source: Front Pharmacol. 2021 Dec 10;12:766293. doi: 10.3389/fphar.2021.766293 (PMC8707065; doi:10.3389/fphar.2021.766293)
Supplement: Supplementary file 7 [file Table2.DOCX]

**Supplementary materials:**

**Table S2: Sporulation characteristics of *C. difficile* clinical isolates.** Isolates were grown anaerobically on Sorbitol MacConkey (SMC) agar for 10 days at 37°C to generate a vegetative cell: spore suspension. Vegetative cells were lysed and removed by a lysis buffer treatment (37°C, 2h), serial washing in cold sterile water and a subsequent heat treatment (60°C, 20 min) to generate a pure spore suspension.

| ***C. difficile* strain** | **Geographic isolation** | **TcdA/TcdB/CDT** | **Ribotype** | **Spore yield ^a^ (x10^9^ CFU/mL)** | **Sporulation efficiency ^b^ (%)** |
| --- | --- | --- | --- | --- | --- |
| 98011 | Liverpool | +/+/+ | 027 | 4.6 ± 0.3 | 82.7 ± 6.9 |
| 108536 | Japan | +/+/+ | 027 | 5.9 ± 0.3 | 75.9 ± 4.9 |
| 108526 | Japan | +/+/- | 018 | 6.7 ± 0.4 | 83.0 ± 5.2 |
| 98220 | Liverpool | -/-/- | 010 | 4.4 ± 0.4 | 71.6 ± 5.7 |
| 1342 | Glasgow | -/-/- | 005 | 8.0 ± 0.1 | 76.3 ± 1.3 |

^a^ Spore yield determined from serial dilutions plated to BHIS agar supplemented with 0.1% w/v sodium taurocholate. After 48h, germinated spores were enumerated by quantifying vegetative colony forming units (CFU). ^b^ Sporulation efficiency was determined by comparing CFU/mL between treated and untreated samples (data expressed as mean ± SEM; for each isolate N=2, n=3). TcdA, toxin A; TcdB, toxin B; CDT, binary toxin.
