# Supplementary material for: Soluble Non-Starch Polysaccharides From Plantain (Musa x paradisiaca L.) Diminish Epithelial Impact of Clostridioides difficile
Source: Front Pharmacol. 2021 Dec 10;12:766293. doi: 10.3389/fphar.2021.766293 (PMC8707065; doi:10.3389/fphar.2021.766293)

Supplementary materials:

Figure S1. Visualisation of purified *Clostridioides difficile* spores. Purification methods involving vegetative cell lysis (2 h), serial washing with cold sterile water and a heat treatment (60°C, for 20 min) were used to generate a pure *C. difficile* spore preparation. Schaeffer and Fulton endospore stain showing spores isolated from *C. difficile* strain 98011, **A)** before and **B)** after purification. Vegetative cells are stained pink and spores are stained green. Spores are indicated by arrows.

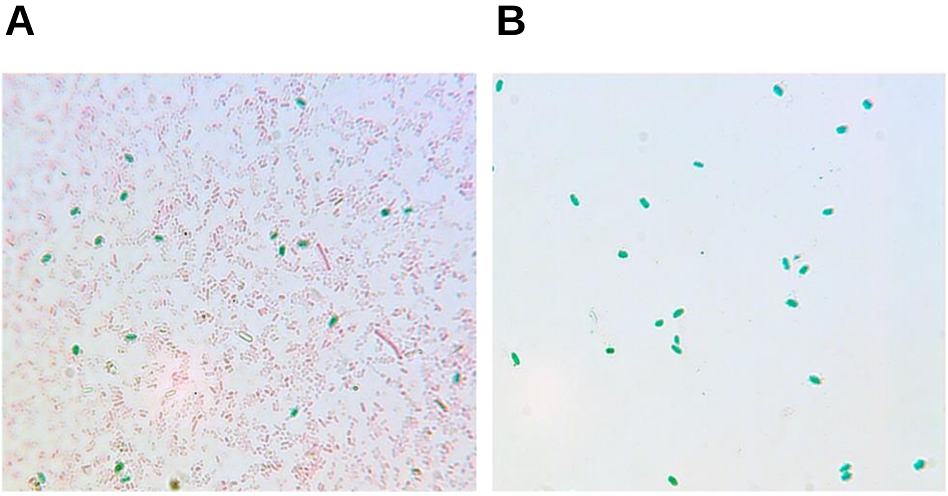

Supplement: Supplementary file 8 [file Image1.pdf]
